# Chromosome-scale assembly with improved annotation provides insights into breed-wide genomic structure and diversity in domestic cats

**DOI:** 10.1016/j.jare.2024.10.023

**Published:** 2024-10-28

**Authors:** Yuki Matsumoto, Claire Yik-Lok Chung, Sachiko Isobe, Mika Sakamoto, Xiao Lin, Ting-Fung Chan, Hideki Hirakawa, Genki Ishihara, Hon-Ming Lam, Shinobu Nakayama, Shigemi Sasamoto, Yasuhiro Tanizawa, Akiko Watanabe, Kei Watanabe, Masaru Yagura, Yoshihito Niimura, Yasukazu Nakamura

**Affiliations:** a Research and Development Section, Anicom Specialty Medical Institute Inc., Yokohama, Kanagawa, Japan; b Data Science Center, Azabu University, Sagamihara, Kanagawa, Japan; c School of Life Sciences and the Center for Soybean Research of the State Key Laboratory of Agrobiotechnology, The Chinese University of Hong Kong, Shatin, Hong Kong Special Administrative Region; d Kazusa DNA Research Institute, Kisarazu, Chiba, Japan; e National Institute of Genetics, Research Organization of Information and Systems, Mishima, Shizuoka, Japan; f Department of Veterinary Sciences, Faculty of Agriculture, University of Miyazaki, Miyazaki, Miyazaki, Japan

**Keywords:** Feline genomics, PacBio sequencing, Hi-C sequencing, Precision medicine, Structural variants, Olfactory receptor genes

## Abstract

**Introduction:**

Comprehensive genomic resources offer insights into biological features, including traits/disease-related genetic loci. The current reference genome assembly for the domestic cat (*Felis catus*), Felis_Catus_9.0 (felCat9), derived from sequences of the Abyssinian cat, may inadequately represent the general cat population, limiting the extent of deducible genetic variations.

**Objectives:**

The goal was to develop Anicom American Shorthair 1.0 (AnAms1.0), a reference-grade chromosome-scale cat genome assembly.

**Methods:**

In contrast to prior assemblies relying on Abyssinian cat sequences, AnAms1.0 was constructed from the sequences of more popular American Shorthair breed, which is related to more breeds than the Abyssinian cat. By combining advanced genomics technologies, including PacBio long-read sequencing and Hi-C- and optical mapping data-based sequence scaffolding, we compared AnAms1.0 to existing Felidae genome assemblies (20 scaffolds, scaffolds N50 > 150 Mbp). Homology-based and ab initio gene annotation through Iso-Seq and RNA-Seq was used to identify new coding genes and splice variants.

**Results:**

AnAms1.0 demonstrated superior contiguity and accuracy than existing Felidae genome assemblies. Using AnAms1.0, we identified over 1.5 thousand structural variants and 29 million repetitions compared to felCat9. Additionally, we identified > 1,600 novel protein-coding genes. Notably, olfactory receptor structural variants and cardiomyopathy-related variants were identified.

**Conclusion:**

AnAms1.0 facilitates the discovery of novel genes related to normal and disease phenotypes in domestic cats. The analyzed data are publicly accessible on Cats-I (https://cat.annotation.jp/), which we established as a platform for accumulating and sharing genomic resources to discover novel genetic traits and advance veterinary medicine.

## Highlights

• AnAms1.0 surpasses felCat9, offering higher accuracy and contiguity in 20 scaffolds.
• AnAms1.0, based on American Shorthair cats, captures a broad range of genetic variations with high accuracy.
• AnAms1.0 reveals over 29 million repetitive variants, 1,500 structural variants and 1,600 novel protein coding genes.
• AnAms1.0 can help identify disease-related variants, thus advancing feline genomic research as well as veterinary care.

## Graphical abstract

**Figure:**
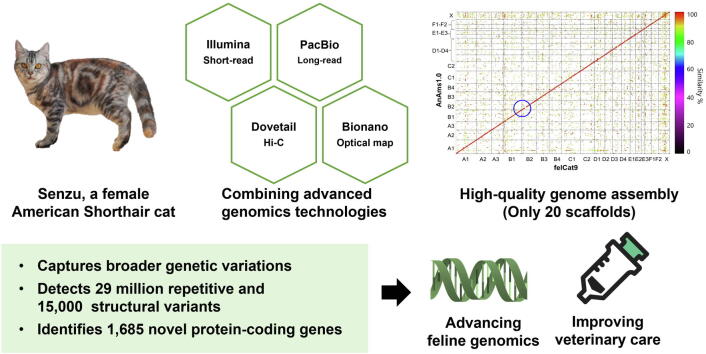

## Introduction

High-throughput sequencing technologies have facilitated the construction of high-quality reference genomes of many domestic animals, including dogs [1] and cats [2]. Studies on these genomes have enabled a deeper understanding of breeding history and trait/disease-related genetic loci, thus promoting advances in veterinary medicine [2], [3], [4], [5].

The domestic cat (*Felis catus*) is one of the most popular companion animals worldwide. Cat domestication began approximately 10,000 years ago in the Middle East [6]. Since then, breeders have developed various cat breeds, and recently, over 40 breeds are recognized by international cat registries, such as The Cat Fanciers’ Association [7]. Modern cat breeds show diverse traits such as coat color, tail length, ear shape, and susceptibility to genetic diseases [8]. As a result, genomic studies can provide valuable insights into breed-specific genes, including those associated with disease phenotypes.

Since the release of the first draft of the domestic cat genome in 2007 [9], subsequent studies on genetic variation in Felidae have rapidly increased, with population genetic structures evaluated using microsatellite DNA markers [10], [11], [12] and genome-wide single-nucleotide polymorphisms (SNPs) [13], [14]. Genetic variation among different breeds has also been extensively investigated. Recent genome-wide studies using high-throughput sequencing have identified breed-specific genetic variations and several candidate genes or regions linked to traits like dwarfism and diseases such as blindness [2], [15], [16], [17], [18], [19]. However, characterizing the genetics and phenotypes of various cat breeds remains challenging due to the absence of a reliable reference genome for many breeds.

The current cat genome assembly, Felis_Catus_9.0 (felCat9), was generated using sequences from a highly inbred Abyssinian cat [20]. The 2017 release of this assembly included significant improvements over the earlier 2014 version [2]. However, as cats have various breeds, multiple high-quality assemblies from different breeds are required to detect genetic variations precisely. Such limitations potentially affect mapping accuracy and cause biases in variant calling and other genomic analyses [21]. To conduct comprehensive research on trait- and disease-associated genetic variations, it is crucial to obtain more high-quality genomes from a broader range of cat breeds [20].

In this study, we constructed a high-quality chromosome-scale assembly, AnAms1.0, from the genome sequences of American Shorthair, one of the most popular cat breeds globally [22]. We selected American Shorthair as the candidate breed for the following reasons: (1) Several genetic markers have revealed that American Shorthair cats have higher heterozygosity than Abyssinians [10], suggesting that the former exhibit higher genome-level variation. (2) Many other popular breeds, such as the Scottish Fold, originated from American Shorthairs, indicating that the latter is commonly used for outcrossing other breeds [11], [22].

## Materials and methods

### Ethics statement

We obtained consent from all cat owners before collecting the biological samples used in this study. The samples were derived from surplus blood collected during routine blood tests, tissues discarded after spay surgeries, or deceased animals. Ethical approval for all sample collection was granted by the relevant ethics committee (Anicom Speciality Medical Institute Inc., 2020-04).

### Animals

**Fig. 1:**
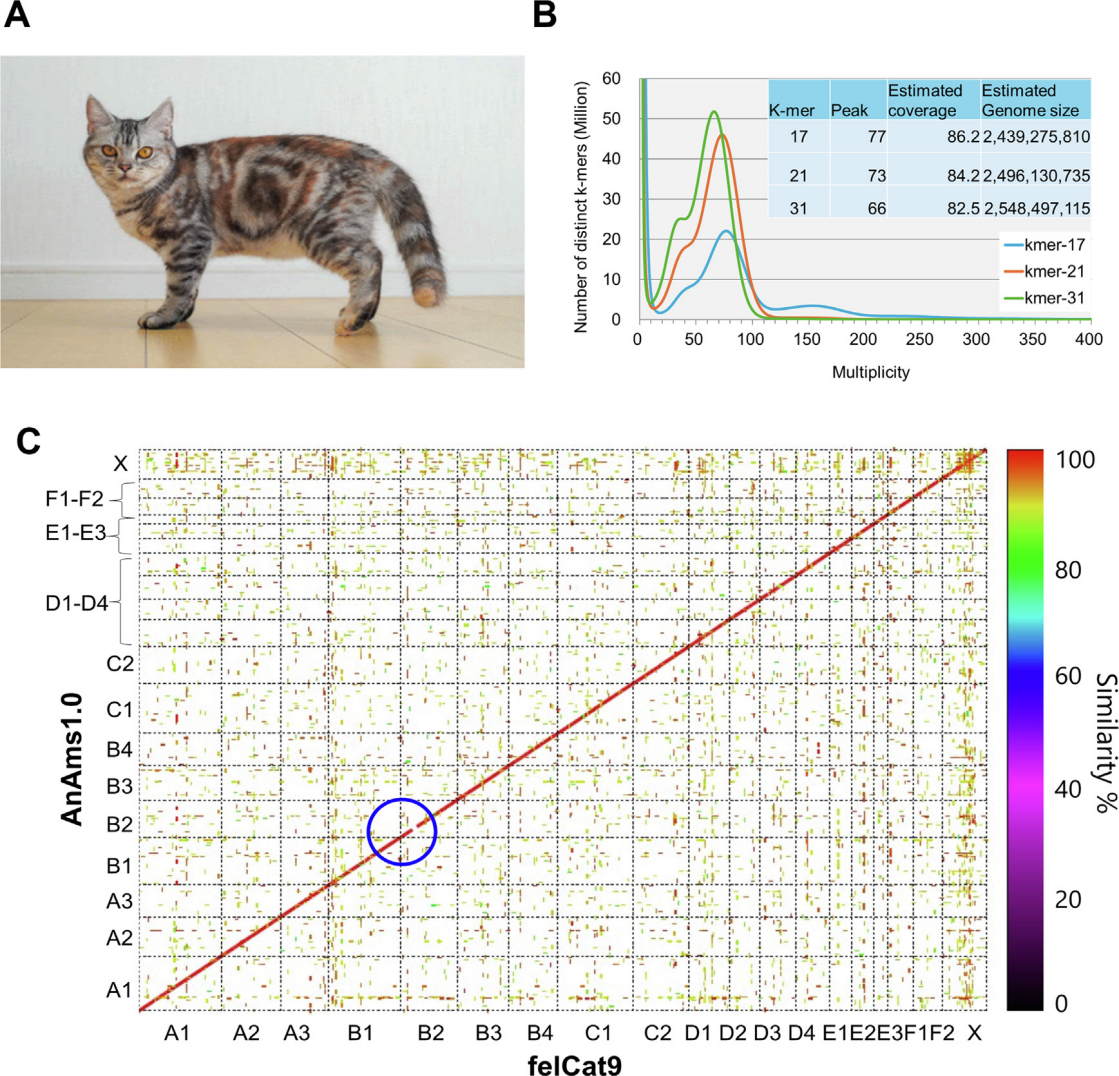
Cat genome assembly using PacBio, Illumina, Hi-C, and OM. (A) Photograph of a female American Shorthair cat named Senzu (Photo: Y. Matsumoto). (B) Distribution of the number of distinct k-mers (k = 17, 21, and 31) in the genome of Senzu with the given multiplicity values. (C) Genome sequence comparison between the 19 chromosomes of AnAms1.0 and felCat9. The blue circle shows incompatible regions on the B2 chromosome. (For interpretation of the references to color in this figure legend, the reader is referred to the web version of this article.).

We selected the individual female American Shorthair cats Senzu and Takae for sequencing after pre-screening using an SNP genotyping array, namely, the Infinium Feline 63 K iSelect DNA array (Illumina, San Diego, CA, USA). The samples were obtained via less invasive swab-based sampling and blood collection. With the owners' consent, we collected blood, ovary, oviduct, and uterine tissue samples from two cats, Senzu and Takae, during their contraceptive surgeries for use in subsequent experiments. Senzu (Fig. 1A) was chosen for genome assembly due to her more inbred genetic background compared to Takae. Samples from Takae were used for gene prediction and annotation through Iso-Seq analysis. For better gene annotation, we also collected tissue samples from four domestic shorthair cat embryos, sourced from Japanese random-bred cats.

### Illumina and PacBio sequencing

Genomic DNA was extracted from Senzu’s blood using a DNeasy Blood and Tissue Kit (Qiagen, Hilden, Germany). Sequence libraries were prepared using SMRTbell Express Template Prep Kit 2.0 (PacBio, Menlo Park, CA, USA). Library size was selected using BluePippin (Sage Science, Beverly, MA, USA) to remove DNA fragments smaller than 30 kb. The library was sequenced on a Sequel system (PacBio) using 28 SMRT cells (PRJDB9879). The sequence reads were assembled using FALCON v.1.8.8 (c) with a length cutoff of 34 kb to generate primary contig sequences and associated contigs representing alternative alleles. Haplotype-resolved assemblies, that is, primary contigs and haplotigs, were generated using FALCON Unzip ver. 1.8.8 [23]. Potential errors in the resulting primary contig sequences were corrected twice using ARROW ver. 2.2.1, implemented on the SMRT Link (v 5.0; PacBio). Paired-end libraries were constructed for Senzu’s DNA and sequenced using an Illumina HiSeqX system (Illumina), with a read length of 151 bp (PRJDB9879). The obtained short reads were used for further error correction of contig sequences using Pilon [24]. Short-read data were also used for genome size estimation using Jellyfish ver. 2.1.4 [25].

### Hi-C scaffolding

A Hi-C library was constructed from an ovary tissue sample collected from Senzu using a Dovetail™ Hi-C Kit with the DpnII restriction enzyme (Dovetail, Sydney, Australia). The library was sequenced using an Illumina HiSeqX to generate 150-bp paired-end reads. The obtained sequence reads were aligned to PacBio contigs for scaffolding using the HiRise pipeline [26].

### Bionano optical map (OM) and hybrid assembly

High-molecular-weight genomic DNA was extracted from the cat uterus using the Bionano Prep Animal Tissue DNA Isolation Kit (Bionano, San Diego, CA, USA), according to the instructions in the Soft Tissue Protocol (Document no. 30,077 Rev C). DNA molecules were then fluorescently labeled using the Bionano Direct Label and Stain protocol. Briefly, flash-frozen tissue was homogenized and embedded into agarose gel to prevent mechanical shearing during nuclear DNA isolation. DNA was recovered from the molten gel via drop dialysis and labeled with the enzyme DLE-1 to produce sequence motif-specific fluorescent signal patterns, followed by YOYO-1 backbone staining to visualize molecule length. Fluorescently-labeled single molecules were stretched, and images were automatically captured in nanochannel arrays in Saphyr Chip G1.2 (Bionano, Part no. 20319) on the Bionano Saphyr platform (Bionano, Part no. 60325). OM data containing molecule length and label distance information were determined from the images using Bionano Access ver. 1.4. We performed *de novo* map assembly using Bionano Solve ver.3.4.1 and Bionano Tools ver. 1.4.1 via Bionano Access v1.4 using the parameters non-human, no preassembly, non-haplotype-aware, no structural variants (SV) mask, and cut complex multipath region. We also performed hybrid scaffolding of the Pilon-polished Hi-C scaffold sequences using Bionano Solve ver. 3.4.1 and Bionano Tools ver. 1.4.1 via Bionano Access v1.4 to resolve conflicts in both the optical map and sequence assemblies.

### Construction of chromosome-level scaffolds

Chromosome-level scaffolds were constructed by aligning the scaffolds with the felCat9 genome (https://www.ncbi.nlm.nih.gov/assembly/GCF_000181335.3/) using RaGOO [27]. The completeness of the assembly was assessed using benchmarking universal single-copy ortholog (BUSCO) ver. 5.4.7 with carnivora_odb10 gene sets, including 14,502 core genes [28]. Genome comparisons were performed using the Nucmer module in MUMmer 3.23 [29] and merqury 1.3 [30]. Known repetitive sequences were registered in Repbase (https://www.girinst.org/repbase/), and *de novo* repetitive sequences defined by RepeatModeler 1.0.11 (https://www.repeatmasker.org/RepeatModeler) were identified using RepeatMasker 4.0.7. (https://www.repeatmasker.org/RMDownload.html).

### Iso-Seq and standard RNA-Seq analysis

Total RNA was extracted from the ovaries, oviducts, and uteri of Senzu and Takae. Quality of the extracted RNA was assessed by using Qubit™ RNA Broad Range (BR) Assay Kits (Thermo Fisher Scientific, Cleveland, OH, USA) and Tape Station (Agilent Technologies, Santa Clara, CA, USA). The RNA was mixed to prepare an Iso-Seq library, which was constructed using the Iso-Seq Template Preparation for Sequel and SMRTbell Template Prep Kit (PacBio), following the manufacturer’s protocol for the Sequel system. The library was sequenced using the Sequel system and two SMRT cells (PRJDB9879). The obtained reads were clustered and quality-filtered using the Iso-Seq 3 pipeline implemented in SMRT Link ver.8.0 (PacBio) mapped to the assembled genome with Minimap2 [31], and then collapsed to obtain non-redundant isoform sequences using a module in Cupcake ToFU (https://github.com/Magdoll/cDNA_Cupcake).

Total RNA was isolated from 12 different tissues (cerebrum, cerebellum, eyeball, heart, lung, liver, spleen, pancreas, stomach, small intestine, large intestine, and kidney) using the NucleoSpin RNA kit (MACHEREY-NAGEL GmbH & Co. KG, Düren, Germany). Short-read sequencing was performed using an Illumina platform, with the sequencing library prepared through the SMART-Seq v4 Ultra Low Input RNA Kit and sequenced on the NovaSeq 6000 system (PRJDB15955).

### Gene prediction and annotation

Gene model predictions were performed as follows: First, the coding sequences (CDSs) of the felCat9 genome (Ensembl release-99, January 2020) and Iso-Seq sequence fragments were mapped to the assembled genome using GMAP (ver. 2019–06–10, [32]). *Ab initio* gene predictions were also performed using BRAKER (v2.1.0) [33], [34], [35] with or without embryo RNA-Seq data. Repeat sequences were annotated using MAKER (v2.31.10) [36]. felCat9 annotations were transferred to AnAms1.0 using Liftoff [37]. The loci to which felCat9 CDS mapped (>95 % of each length), the loci to which Iso-Seq contigs mapped (>97 % of each length), and the loci predicted by *ab initio* were added to create tentative gene models (Fig. S1). Then, we performed manual curation with Apollo [38]. Our curation process included the following steps: (1) We first discarded gene models with excessively long introns, particularly those containing other gene models in the same direction. (2) For felCat9 CDS with different IDs at the same locus, we compared the synteny between felCat9 and AnAms1.0, ensuring they matched felCat9′s synteny. (3) We added Iso-Seq gene models to loci lacking a felCat9-derived gene model. The final protein-coding gene candidate loci were translated into proteins using GffRead [39]. Functional annotation was conducted with InterProScan (v5.50–84.0 [40]), the KEGG Automatic Annotation Server (KAAS [41]), and RPS-BLAST [42]. The felCat9 genome annotation was retrieved from Ensembl release-99 (Jan 2020). Repetitive sequences in the AnAms1.0 D2:45000000–55000000 region were identified by analyzing the corresponding coordinates of the D2 chromosome in felCat9 (*Felis_catus*.Felis_catus_9.0.dna.chromosome.D2.fa) and AnAms1.0 were extracted using seqkit [43] and aligned using MUMmer. The regions with repetitive structures were identified by visual inspection.

### Olfactory receptor (OR) gene analysis

Intact OR genes and OR pseudogenes were identified in the AnAms1.0 and felCat9 sequences using a previously described method [44], with modifications [45]. The correspondence of OR genes between AnAms1.0 and felCat9 was determined as follows: we calculated the amino acid sequence identity between an AnAms1.0 OR gene and a felCat9 OR gene for all possible pairwise comparisons to extract the reciprocal best hits. Among the reciprocal best hits, OR gene pairs showing > 97 % amino acid sequence identity were considered as corresponding (Table S1).

### Base and structure variants against felCat9

Base variants (SNPs and Indels) and SVs of the assembled genome were compared with those of the felCat9 genome. To detect base variants, the Illumina PE reads of Senzu were mapped to both the assembled genome and the felCat9 genome using Bowtie2 [46]. Moreover, felcat short reads (SRR5055389) were mapped onto the assembled and the felCat9 genomes. Variant calls were performed using bcftools 1.9 in SAMtools [47]. Possible false base variants were filtered out under the following conditions: exclude multiple alleles, minimum DP = 20, maximum DP = 100, minimum GQ = 50, minimum QUAL = 200, and max-missing = 1.0. SVs were identified using the pbsv module in SMRT Link 6.0 (PacBio).

Whole-genome sequencing (WGS) data from 100 cats, including 94 domestic cats (58 from 15 inbred and 36 random-bred from four regions: USA, Europe (EUR), Middle East/Africa (MEA), and Asia) and 6 wild cats, were analyzed to compare mapped reads and variant numbers. Blood or tissue samples were collected from 29 cats that visited an animal hospital. Sequencing libraries for these 29 samples were generated using the TruSeq DNA PCR-Free Kit (Illumina) and sequenced on a NovaSeq 6000 platform (PRJDB15975). An additional 71 WGS datasets were downloaded from the Sequence Read Archive (Table S2). Low-quality reads (Phred score < 30), adapter sequences, and reads shorter than 50 nucleotides were removed using Trim_galore (https://github.com/FelixKrueger/TrimGalore). Quality-filtered reads were then mapped and aligned to AnAms1.0 and felCat9 reference genomes using DRAGEN v4.0.3 (Illumina Inc.) with the enable-map-align method. The output was obtained as gvcf files, which were then merged using the DRAGEN enable-joint-genotyper option. Default settings were applied for filtering genotype calls.

The numbers of mapped reads and called variants for AnAms1.0 and felCat9 were compared. DRAGEN was used to generate the numbers of mapped reads and variants, which were then compared. RTG tools calculated the number of SNPs, insertions, deletions, and structural variants. We used R software v4.2.3 to calculate the ratio of mapped reads, and t-tests were performed to compare the number of variants between the assemblies.

### Stranded RNA-Seq for lncRNA prioritization using an integrated approach

Approximately 400 ng of total RNA from blood samples was enriched for poly-A RNA using the NEBNext® Poly(A) mRNA Magnetic Isolation Module (NEB #E7490; New England Biolabs [NEB], Ipswich, MA, USA) or depleted of rRNA using the NEBNext® Globin & rRNA Depletion Kit (Human/Mouse/Rat, NEB #E7750). Stranded RNA-Seq libraries were prepared using either the NEBNext® Ultra™ II Directional RNA Library Prep Kit for Illumina® (NEB #E7760) or the SEQuoia Complete Stranded RNA Library Prep Kit (17005726; Bio-Rad Laboratories, Hercules, CA, USA), following the manufacturer’s instructions. Four libraries were prepared using different RNA enrichment methods and library preparation kits. The RNA-Seq libraries were sequenced at Novogene on the NovaSeq 6000 platform (Illumina), and the data (Fig. S2A) were deposited in the NCBI BioProject database (BioProject accession number PRJNA721786, https://www.ncbi.nlm.nih.gov/bioproject/).

Quality control and adapter trimming were performed differently depending on the kit used. For libraries prepared with NEB kits, Trimmomatic v0.39 [48] was used with the parameters: “ILLUMINACLIP: adapters.fa:2:30:10 SLIDINGWINDOW:4:28 MINLEN:50,” trimming the adapter sequences 5′-ACACTCTTTCCCTACACGACGCTCTTCCGATCT-3′ and 5′-GATCGGAAGAGCACACGTCTGAACTCCAGTC-3′. For the Bio-Rad libraries, cutadapt v3.3 was used with the parameters suggested by the manufacturer: “-u 1 −a A{10} −m 15”.

Clean reads were used for both reference-based and de novo transcript assemblies. In reference-based assembly (Fig. S2B), clean reads were mapped to the reference genome using HISAT2 v2.2.1 [49], followed by transcript assembly with StringTie2 v2.1.4 [50]. For de novo transcript assembly (Fig. S2C), clean reads were assembled using Trinity v2.11.0 [51] and mapped to the reference genome using GMAP v2020.10.14 [32]. In addition, 26,662 full-length Iso-Seq transcripts (Pacific Biosciences, Menlo Park, CA, USA) were mapped to the reference genome using Minimap2 v2.17 [31] and collapsed into representative transcripts using the NIAP_v1.1.pl script in NIAP (https://github.com/alanlamsiu/NIAP, Fig. S2D). Transcripts from the reference-based assembly and representative Iso-Seq transcripts were merged using the NIAP_merge_v1.1.pl script (Fig. S2B & D). These merged transcripts were annotated to the reference genome using the NIAP_annotate_v1.4.pl script (Fig. S2E). Non-coding transcripts predicted by infernal v1.1.4 [52] using the Rfam database v14.3 [53] were included in the annotation.

Loci annotated as antisense, intronic, intergenic, or overlapping were considered unannotated, and their transcripts were prioritized as lncRNAs in two steps. In step 1 (Fig. S2F), unannotated loci were filtered if they contained any transcript that (1) was shorter than 200 nt, (2) had a coding potential above 0.364 for humans or 0.44 for mice as predicted by CPAT v3.0.3 [54], or (3) had a high-scoring segment pair with an e-value ≤ 0.1 in a blastx search (v2.10.1) [55] against the NCBI non-redundant protein database. In step 2 (Fig. S2G), the remaining transcripts were compared with de novo assembled transcripts using the NIAP_compare_v1.1.pl script. Only transcripts supported by Iso-Seq data or de novo assemblies were retained in the final set of high-confidence lncRNAs.

### Analysis of lncRNA conservation

LncRNA transcripts were mapped to the human reference genome (GRCh38.p13) using GMAP. The best alignment was used for calculating a conservation score as follows: conservation score = identity × coverage × 10^-4^. The average phastCons and phyloP scores across 100 mammalian species of the aligned regions in the human genome for each transcript were calculated using UCSC data (https://hgdownload.soe.ucsc.edu/goldenPath/hg38/) and utilities (https://hgdownload.cse.ucsc.edu/admin/exe/). As a control, protein-coding transcripts were aligned to the human reference genome to calculate the aforementioned scores. Sequence alignments were generated by Clustal Omega [56] and visualized using Jalview [57]. Human gene annotations from GENCODE v37 were used [58].

### Functional analysis of antisense and intronic lncRNA loci

Protein-coding genes overlapping antisense lncRNA loci on the opposite strand and intronic ones on the same strand were identified using the “intersect” function of BEDTools [59]. The associated Gene Ontology and KEGG pathway terms were extracted from the genome annotation, tabulated, and then ranked by the number of genes belonging to each term.

### SV analysis of OM data

SVs were identified by comparing AnAms optical maps with felCat9 and AnAms1.0 genomes using Bionano Solve (v3.5; Bionano Genomics; CA, USA) and the runBNG wrapper (v2.0.1) [60]. Insertions, deletions, and inversions with a confidence score < 0.5 were filtered out. Translocation calls that were not present in the primary mapping were also removed. Genomic coordinates of SV results against the felCat9 genome were converted to match those in AnAms1.0 to remove SVs commonly called in equivalent regions in both genomes. Briefly, a “chain” file that denotes the coordinate relations between the two genomes was generated using pblat (v2.5) [61] alignment and UCSC tools. Block coordinate conversion was performed using the region module in CrossMap (v0.6.1) [62]. Functional annotations of SV regions were obtained using BEDTools (v2.29.2) [59] intersected with genome annotations of AnAms1.0 for insertions and felCat9 for deletions. Enrichment analysis of the affected gene annotations was performed using DAVID (v6.8) [63] and ingenuity pathway analysis (IPA) (v70750971, release Oct 2021) [64]. A total of 10,798 of the 13,703 insertions and 2,454 of the 2,630 annotated genes had IDs successfully mapped to the database for analysis. Owing to the maximum number of 8,000 input genes, IPA variant analysis was performed only on genes affected by deletions, which included 602 unique gene IDs mapped to the database. Enriched Gene Ontology terms, KEGG pathways, and canonical pathways were recorded.

### Population genomic analysis

WGS data described in the “Base and structure variants against Felis_catus_9.0” section was subjected to the following population genomic analysis. We used Plink v 1.9 [65] to convert the binary files (bed/bim/fam) from a joint-vcf file. ADMIXTURE [66] analysis was done by using the converted binary files to infer the ancestry, and the population *K* with the lowest cross-validation errors was used. Following fasta generating using plink ped was used for reconstructing phylogeny of a neighbor-joining method with 1,000 bootstraps by using Splitstree v6 [67].

## Results

### Genome assembly construction

A total of 1.54 G reads were obtained from an Illumina Paired-End library generated from the sequences of a female American Shorthair cat named Senzu (Fig. 1A). The distribution of distinct k-mers (k = 17) showed two peaks at multiplicities of 77 and 154, projecting an estimated genome size of 2.44 and 1.22 Gb, respectively (Fig. 1B). The estimated genome size with distinct k-mers of k = 17, 21 and 31 were 2.44, 2.50, and 2.55 Gb, respectively (Fig. 1B). Our estimation was smaller than the previous study of 2.7 Gb [9] but close to the total length of the felCat9 genome (Table 1).

### Genome assembly

A total length of 345.4 Gb of PacBio reads was generated from 28 SMRT cells, and read lengths of ≥ 35 kb were used for assembly. The total length and coverage of the Senzu genome (2.44 Gb) were 120.7 Gb and 49.5×, respectively. The FALCON Unzip assembly generated 547 primary and 5,512 haplotig contigs. The genome sequences of the primary contigs were scaffolded with N100 based on Hi-C reads after error correction using PacBio and Illumina reads.

A total of 1,225,034 OM molecules at least 150 kb long, labeled at DLE-1 enzyme recognition motifs, were obtained from the Bionano Saphyr run, with an N50 and total length of 336,375 bp and 383.6 Gb, respectively (Fig. S3A). The molecules were assembled into 118 genome maps containing 2,419 Mbp, with a chromosome-level N50 length of 73.1 Mbp (Fig. S3B). In sequence scaffolding, a total of 372 sequence scaffolds (see supplemental information) were input for OM modification, of which 87 sequences were joined into 37 super-scaffolds containing 2.46 Gb, i.e., 99.4 % of the input sequences (Table S3 and Fig. S4). In the process, a total of 13 misassemblies were detected by mapping the OM scaffolds, where Hi-C scaffolds were split. Decreases in the scaffold N50 and maximum length resulted from the resolution of assembly conflicts, indicating the correction of misassemblies.

Chromosome-level scaffolding was performed by aligning the 348 scaffold sequences modified via OM with the felCat9 genome using RaGOO. The resultant sequences were designated as AnAms1.0 (Table 1, S3, and S4). AnAms1.0 consists of 19 chromosome-level scaffolds and only one short unmapped scaffold, which was 1,381 bp long. The 19 chromosome-level scaffolds represent 18 autosomal and one sex (X) chromosome. The corresponding chromosomal numbers were determined based on the felCat9 genome sequence. The total length of AnAms1.0 is 2,493,141,643 bp, and the N50 was 151 Mbp, which is the longest N50 in the six feline genome assemblies.

Investigation of the assembly quality of AnAms1.0 by mapping sequences to 14,502 BUSCOs revealed 13,832 (95.4 %) complete BUSCOs, including 13,670 (94.3 %) single-copy genes and 162 (1.1 %) duplicated genes (Table 2). The number of fragmented and missing BUSCOs was 122 (0.8 %) and 548 (3.8 %), respectively. The number of complete BUSCOs was the largest in AnAms1.0 (13,832) and lowest in PanTig1.0 (13,081). Fragmented and missing BUSCOs were the lowest in AnAms1.0 among the six assemblies (122 and 548, respectively). For repetitive sequences, we found 880 million variants in AnAms 1.0 and thus over 29 million repeats differed from felCat9.

**Table 1:** Assembly statistics for six feline genome assemblies.

Assembly	Scientific name	Common name	Total length	Number of scaffolds	Scaffold N50 (Mbp)	QV	Error rate	Percent gaps (%)
AnAms1.0	*Felis catus*	Domestic cat	2,493,141,643	20	151	45.8	0.00003	0.008
felCat9	*Felis catus*	Domestic cat	2,521,710,482	4,507	149	30.1	0.00098	0.018
F.catus_Fca126_mat1.0*	*Felis catus*	Domestic cat	2,425,747,038	71	148	41.7	0.00007	0
PanLeo1.0	*Panthera leo*	Lion	2,406,807,619	8,060	136	42.4	0.00006	0.008
PanTig1.0	*Panthera tigris altaica*	Amur tiger	2,391,065,193	1,478	8	11.5	0.07157	0.024
PanPar1.0	*Panthera pardus*	Leopard	2,578,002,243	50,376	21	43.5	0.00005	0.038

* QV and Error rate were reffered from Bredemeyer et al. **(2023).**

**Table 2:** BUSCO score for six feline genome assemblies.

**Assembly**	**Complete BUSCOs (%)**	**Complete and single-copy BUSCOs (%)**	**Complete and duplicated BUSCOs (%)**	**Fragmented BUSCOs (%)**	**Missing BUSCOs (%)**
AnAms1.0	13,832 (95.4)	13,670 (94.3)	162 (1.1)	122 (0.8)	548 (3.8)
felCat9	13,789 (95.1)	13,661 (94.2)	128 (0.9)	136 (0.9)	577 (4.0)
F.catus_Fca126_mat1.0	13,826 (95.3)	13,718 (94.6)	108 (0.7)	124 (0.9)	552 (3.8)
PanLeo1.0	13,683 (94.4)	13,553 (93.5)	130 (0.9)	161 (1.1)	658 (4.5)
PanTig1.0	13,081 (90.2)	12,998 (89.6)	83 (0.6)	445 (3.1)	976 (6.7)
PanPar1.0	13,776 (95.0)	13,640 (94.1)	136 (0.9)	141 (1.0)	585 (4.0)

Total 14,502 BUSCO groups searched in carnivora_odb10 datasets (BUSCO v5.4.7).

A graphical view of syntenic relationships between AnAms1.0 and felCat9 for the 19 scaffolds is shown in Fig. 1C. Alignment of homologous sequence pairs along each scaffold revealed high similarity between the two genomes, except for the B2 chromosome.

### Repetitive sequences

**Fig. 2:**
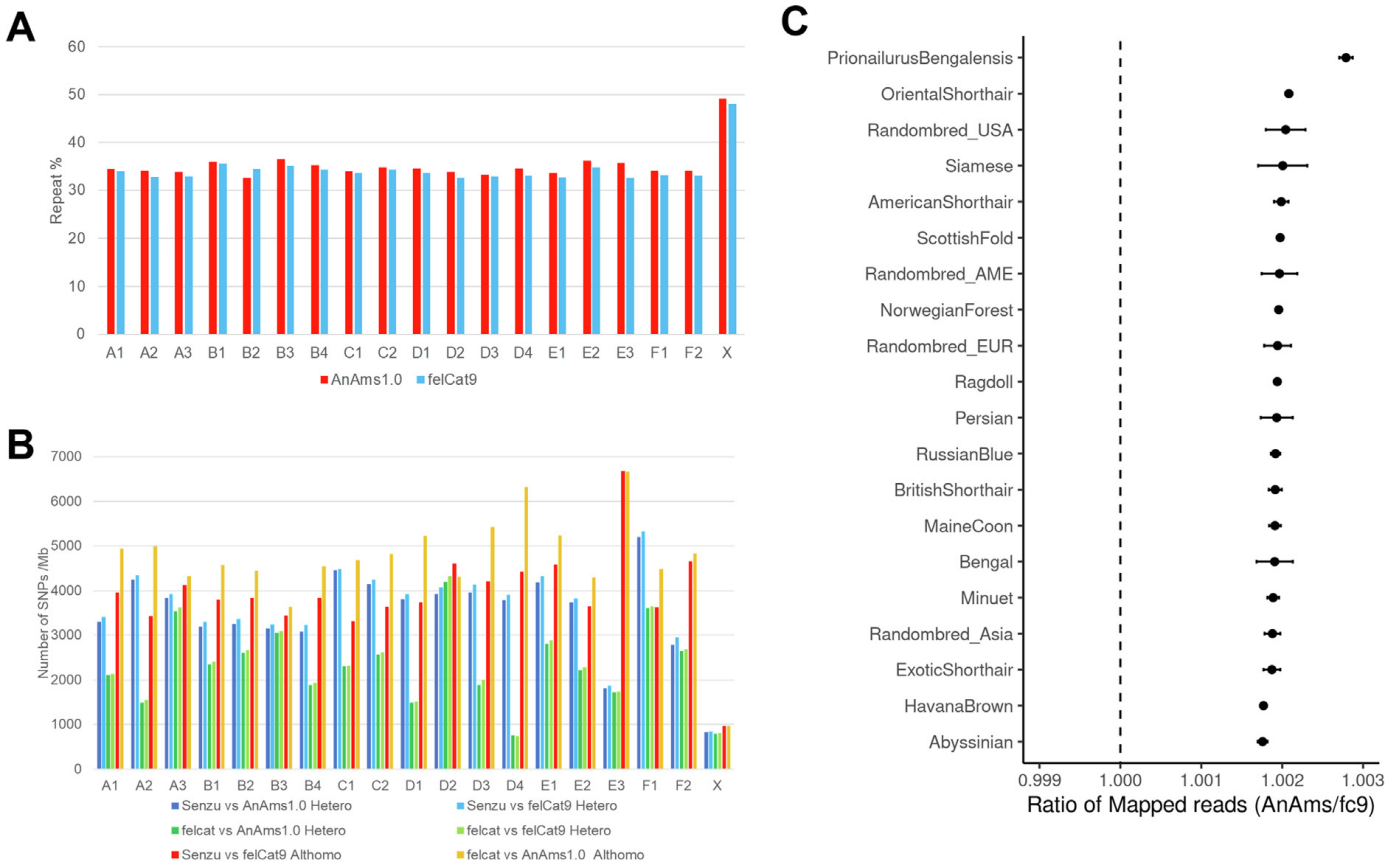
Comparison of variants called between AnAms 1.0 and felCat9. (A) Percentages of repetitive sequences on each chromosome-scale scaffold. (B) Average density of base variants identified by mapping Illumina reads to each chromosome of AnAms1.0 and felCat9. (C) Ratio of mapped reads among 15 cat breeds, four random-bred populations and a wild leopard cat, *Prionailurus Bengalensis* (n > 3) obtained using short-read data.

The total length of repetitive sequences in AnAms1.0 was 880.2 Mbp, accounting for 35.3 % of the assembled genome (Table S5). The ratio of repeat sequences was similar to that in felCat9 (34.6 %). The percentage of repetitive sequences on each chromosome-level scaffold ranged from 32.6 % (B2) to 36.6 % (B3) in the autosomal chromosomes of AnAms1.0 but was 49.1 % in the X chromosome (Fig. 2A).

### Detection of genetic variants via comparison with felCat9

The paired-end (PE) reads from Senzu and Cinnamon were aligned to their respective assembled sequences, and detected variants were filtered to remove those in repetitive regions. Mapping Senzu's Illumina PE reads to the AnAms1.0 genome assembly resulted in the detection of 2,654,090 variants. In contrast, mapping Cinnamon's reads to the felCat9 genome yielded 5,550,296 variants. Of these, 1,642 homozygous alternate alleles were found on AnAms1.0, while 2,818,968 were detected on felCat9. The presence of homozygous alternate alleles suggests possible misassemblies. The smaller number of such variants on AnAms1.0 compared to felCat9 indicates that AnAms1.0 may have a higher assembly accuracy.

The variant density, calculated by dividing the number of variants by the length of masked sequences, varied across chromosomes (Fig. 2B). Both Senzu and felCat9 exhibited a low density of variants on the X chromosome, whereas the variant densities on other chromosomes differed between Senzu and felCat9, suggesting that the X genome had lower heterozygosity, whereas the heterozygosity of other regions varied depending on breeds or individuals. The low density of alt homo variants on the X chromosome also indicated a higher sequence similarity between Senzu and felCat9 in the X chromosome compared with that in other chromosomes.

We mapped Illumina short-read data from 100 domestic and wild cats to AnAms1.0 and compared the assembly to that in felCat9. The average number of variants called in the whole genome was 12,668,341 (1,114,677 (SD)), 13,420,788 (802,748 (SD)) and 35,780,076 (2,555,352 (SD)) in domestic (inbred), domestic (random-bred) and wild cats, respectively. In a comparison of short-read data from 15 cat breeds, including the Abyssinian breed on which felCat9 is based, the number of mapped reads in AnAms1.0 exceeded that in felCat9 (Fig. 2C). Furthermore, the total number of called SNPs, insertions, and deletions was higher in felCat9 than in AnAms1.0 for all pairs without Abyssinian (Fig. S5, Table S6).

To identify SVs, we mapped Senzu PacBio reads to the felCat9 genome. A total of 16,035,301 PacBio sub-reads were mapped across the felCat9 genome, with a mean coverage of 120.1×. The number of identified SVs was 155,535, with a total length of 45,222,456 bp (Table S7). The most frequently observed SVs were deletions (55.1 %) and insertions (39.3 %).

The distribution of mapped PacBio read depth showed large insertions and deletions in the Senzu genome against felCat9 (Fig. S6). Possible insertions were observed on most chromosomes. A large deletion was identified on the E3 chromosome. The large deletion identified on the B2 chromosome using Nucmer was not detected via SV analysis.

We further investigated SVs between the Senzu and felCat9 genomes using optical maps (Fig. S7). By mapping the assembled optical maps from Senzu to *in silico*-digested maps from the felCat9 and AnAms1.0 sequences, 10,180 and 1,090 SVs were called, respectively. Removal of regions commonly called in both assemblies to avoid false positives, such as those arising from gaps, via coordinate conversion and intersection yielded a total of 134,480 genomic regions. The potential functional impact of these SV candidates was analyzed via enrichment analysis using DAVID (Fig. S8). Clusters of terms related to cardiomyopathy were enriched, consistent with IPA results (Fig. S9). In addition, “nitric oxide signaling in the cardiovascular system” and “drug response to nitric oxide-mediated vasodilator” were the enriched terms in the IPA of variants.

### Iso-Seq analysis

Iso-Seq sequences totaling 20.9 and 31.6 Gb in length were obtained from two American Shorthair cats, Senzu and Takae, respectively. The sequences from the two individual cats were integrated and clustered using Iso-Seq3, whereafter, the high-quality (hq) sequences were collapsed and filtered using Cupcake ToFU. The total number of resultant sequences decreased from 50,436 (Iso-Seq3/hq) to 26,662 (collapsed/filtered) during the assembly process (Table S8). The ratio of complete BUSCOs showed no significant difference between the IsoSeq3/hq, collapsed, and collapsed/filtered results. The percentage of complete BUSCOs ranged from 46.7 % to 47.1 %, while the proportion of missing BUSCOs varied from 40.8 % to 41.3 %. In the Iso-Seq analysis, transcript sequences were derived from the ovary, oviduct, and uterus (Table S8). The high proportion of missing BUSCOs is likely due to the limited number of organs sampled.

### Gene prediction and annotation

We performed gene prediction and annotation (Fig. S1). Of the 19,587 protein-coding genes in the felCat9 annotation (Ensembl release-99, January 2020), 19,562 genes were mapped to AnAms1.0. Of the 26,662 sequences obtained with Iso-Seq, 26,614 were mapped to AnAms1.0. The mapping results of felCat9 transcripts, Iso-Seq sequences, and manual curation identified 21,272 loci in AnAms1.0 (Table 3). Repeat sequence prediction using MAKER, along with mapping felCat9 annotations through Liftoff and loci prediction via BRAKER, supported these results. Out of the 21,272 loci identified, 18,703 were manually curated, while 2,569 loci, although not manually curated, were validated as CDS-mapped loci from felCat9. (Table S9). The number of protein-coding genes on each chromosome was compared with that of felCat9, and the number of protein-coding genes annotated with AnAms1.0 was higher than that with felCat9 for all chromosomes despite not containing any mitochondrial genes (Table S10). AnAms1.0 contained 21,272 gene loci (1,685 more than those in felCat9; Table 3). The higher number of predicted genes in AnAms1.0 is primarily attributed to (1) Genes located on unplaced contigs in felCat9 were mapped to one of the chromosomes in AnAms1.0; (2) Some genes had an increased copy number in AnAms1.0 compared with that in felCat9. Further details regarding the manually curated gene models can be found in Table S11.

**Table 3:** Comparison of AnAms1.0 with felCat9 genome annotations.

**Feature**	**AnAms1.0**	**felCat9**
genes	21,272	19,587
genes (chromosomal)	21,272	19,376
mRNAs	37,918	40,279

felCat9 annotation is from Ensembl release-99(Jan 2020) and other Felidae annotations are from Ensembl release-103(Feb 2021). AnAms1.0 does not include the mitochondrial genome. All features were enumerated using an in-house R script from each gff-formatted annotation.

Protein domain annotations of the translation products of these loci were obtained using InterProScan, KAAS, and RPS-BLAST. Gene categorization according to their functional ortholog groups revealed many genes related to signal transduction, particularly olfactory transduction. Additionally, many genes were related to transcriptional and post-translational modifications (Fig. S10). Other details on functional annotation are provided in Appendix (See Supporting data).

**Fig. 3:**
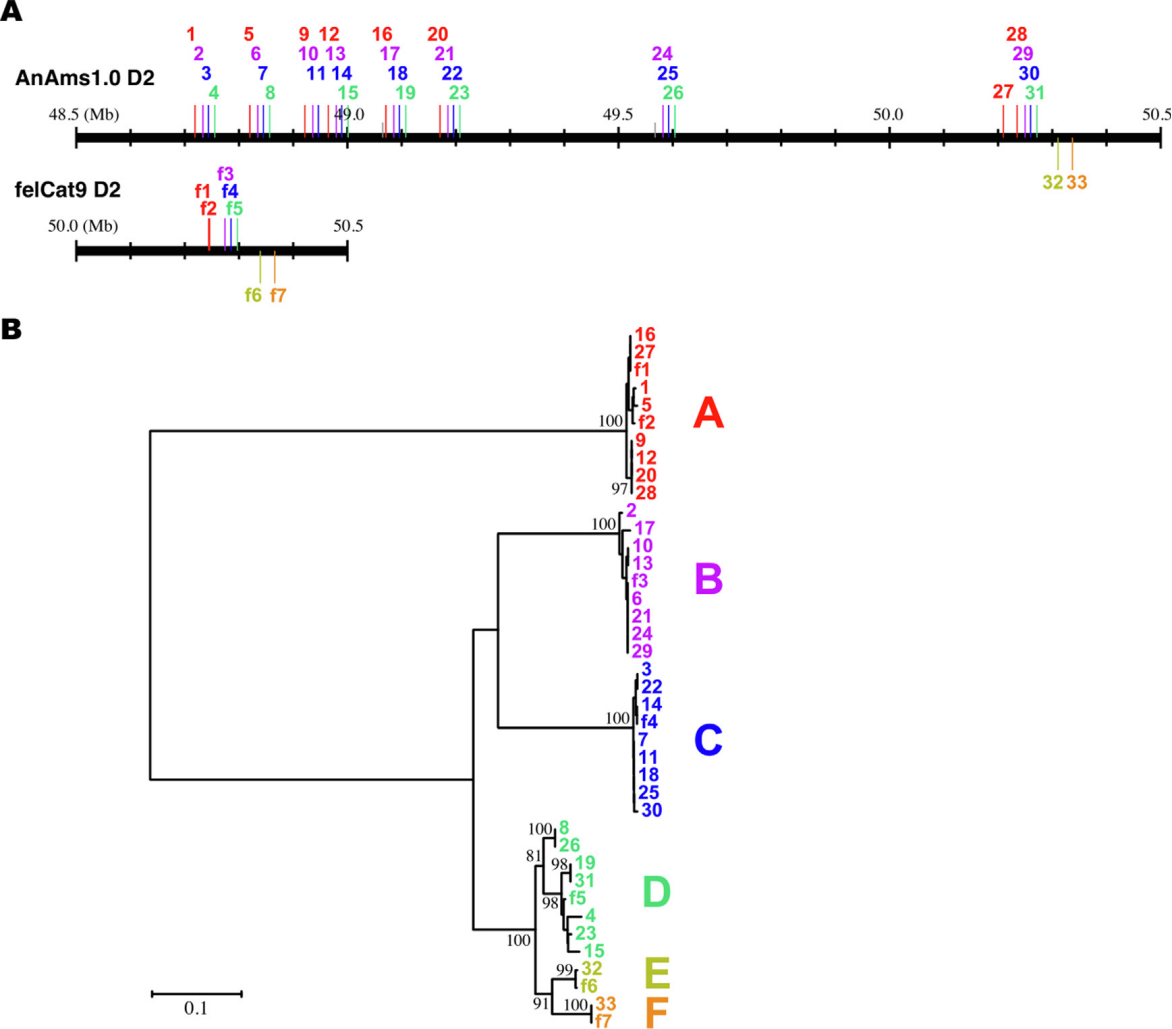
Genomic and phylogenetic characterization of chromosome D2 between AnAms 1.0 and felCat9. (A) Comparison of the genomic regions of AnAms1.0 D2:48500000–50500000 and felCat9 D2:50000000–50500000. The long and short vertical bars represent the genomic locations of an intact olfactory receptor (OR) gene and an OR pseudogene, respectively. In the AnAms1.0 genomic region, 33 intact OR genes are numbered along the chromosome and colored according to their phylogenetic clades shown in (B). This region also contains two pseudogenes (shown in gray, without numbers). The felCat9 genomic region contains seven intact OR genes, named f1–f7 and colored according to (B). (B) Neighbor-joining phylogenetic tree for the 33 and seven intact OR genes in AnAms1.0 D2:48500000–50500000 and in felCat9 D2:50000000–50500000, respectively. Gene names are as indicated in (A). Phylogenetic clades A–F, clustered with a 95 % amino acid identity threshold, are colored differently. Bootstrap values obtained from 500 resamplings are shown for the nodes with >80 % bootstrap support. The scale bar indicates the number of amino acid substitutions per site.

Curation of the gene model revealed the existence of a region on chromosome D2 of AnAms1.0, where the same CDS from felCat9 was mapped to multiple loci. Therefore, we aligned the D2 chromosome of AnAms1.0 with felCat9 using MUMmer and examined the sequence homology. A repetitive region with a maximum length of approximately 100 kb was identified in AnAms1.0 D2:48700000–49200000, and regions with similar characteristics were identified on other chromosomes. This repetitive region was not detected in the felCat9 sequence (Fig. S11) and contained OR genes (Fig. 3).

OR genes form the largest multigene family in mammals and are dispersed as multiple genomic clusters across many chromosomes [68]. To comprehensively investigate the genomic distribution of OR genes, we identified all OR genes in AnAms1.0 and felCat9 sequences. AnAms1.0 contained a significantly larger number of intact OR genes (902) than felCat9 (768) (Tables S1 and S12). Of the 768 intact OR genes in felCat9, 25 were located in a short contig that did not assemble into a single chromosome. The D2:48500000–50500000 region of AnAms1.0 contained 33 intact OR genes, whereas the corresponding region of felCat9, D2:50000000–50500000, contained only seven (Fig. 3A). The AnAms1.0 genomic region contains eight repeat units (1–4, 5–8, 9–11, 12–15, 16–19, 20–23, 24–26, and 28–31), each comprising of four (or three) intact OR genes from phylogenetic clades A–D (Fig. 3B), whereas the felCat9 region contains only one repeat unit. The presence of the repeats in AnAms1.0 was also depicted in Fig. S11B. Furthermore, in several cases, part of an OR genomic cluster was missing from the felCat9 sequence or was encoded in unassembled short contigs (Fig. S12). The results of the read-based assessment showed that D2:50000000–50500000 in felCat9 showed high coverage, suggesting that the region in felCat9 has collapsed. On the other hand, in AnAms1.0, no individual showed disproportionately high coverage, although OR gene-clustered regions showed different patterns in individuals (Fig. S13). There may be individual variation around the OR gene on chromosome D2. Further analysis with another technique, such as ultra-long reads, is needed to clear this region. These observations suggest that the genome assembly quality was significantly improved in AnAms1.0 compared with that in felCat9.

### Conservation analysis and functional annotation of predicted lncRNA genes

To increase the transcriptome coverage for predicting lncRNA genes, we used deep short-read RNA-Seq data from the blood total RNA of Senzu to construct four RNA-Seq libraries using different RNA enrichment methods and sequencing kits, generating at least 42 million read pairs per sample (Fig. S2A). We employed an integrated assembly method, wherein reference-based (Fig. S2B) and reference-free (Fig. S2C) *de novo* transcriptome assemblies were performed for each library (Fig. S2D). The four reference-based assemblies were merged with transcripts consolidated from 26,662 Iso-Seq transcripts (Fig. S2D) and annotated using the protein-coding annotation. In total, 15,844 unannotated loci, containing 35,102 transcripts, were identified (Fig. S2E). The coding properties of these transcripts were assessed by calculating the coding probability and searching against the NCBI non-redundant protein dataset (Fig. S2F). After filtering based on length, coding probability, and matches in the non-redundant protein database, 13,140 transcripts from 9,086 loci were identified as potential lncRNAs (Fig. S2F). Of these, 7,623 transcripts from 6,036 loci were supported by the Iso-Seq data or the reference-free assemblies and thus could be used as high-confidence lncRNAs for downstream analyses (Fig. S2G).

A set of high-confidence lncRNAs was further classified based on their genomic context. Most sequences were classified as antisense (2,017 loci, 2,862 transcripts) and intergenic (2,496 loci, 2,869 transcripts) lncRNA genes, followed by overlapping (1,066 loci, 1,425 transcripts) and intronic (457 loci, 467 transcripts) ones (Fig. S2H). For further details of lncRNA profiles, see Supporting information.

### Genome-wide variant profiling and population genomics

**Fig. 4:**
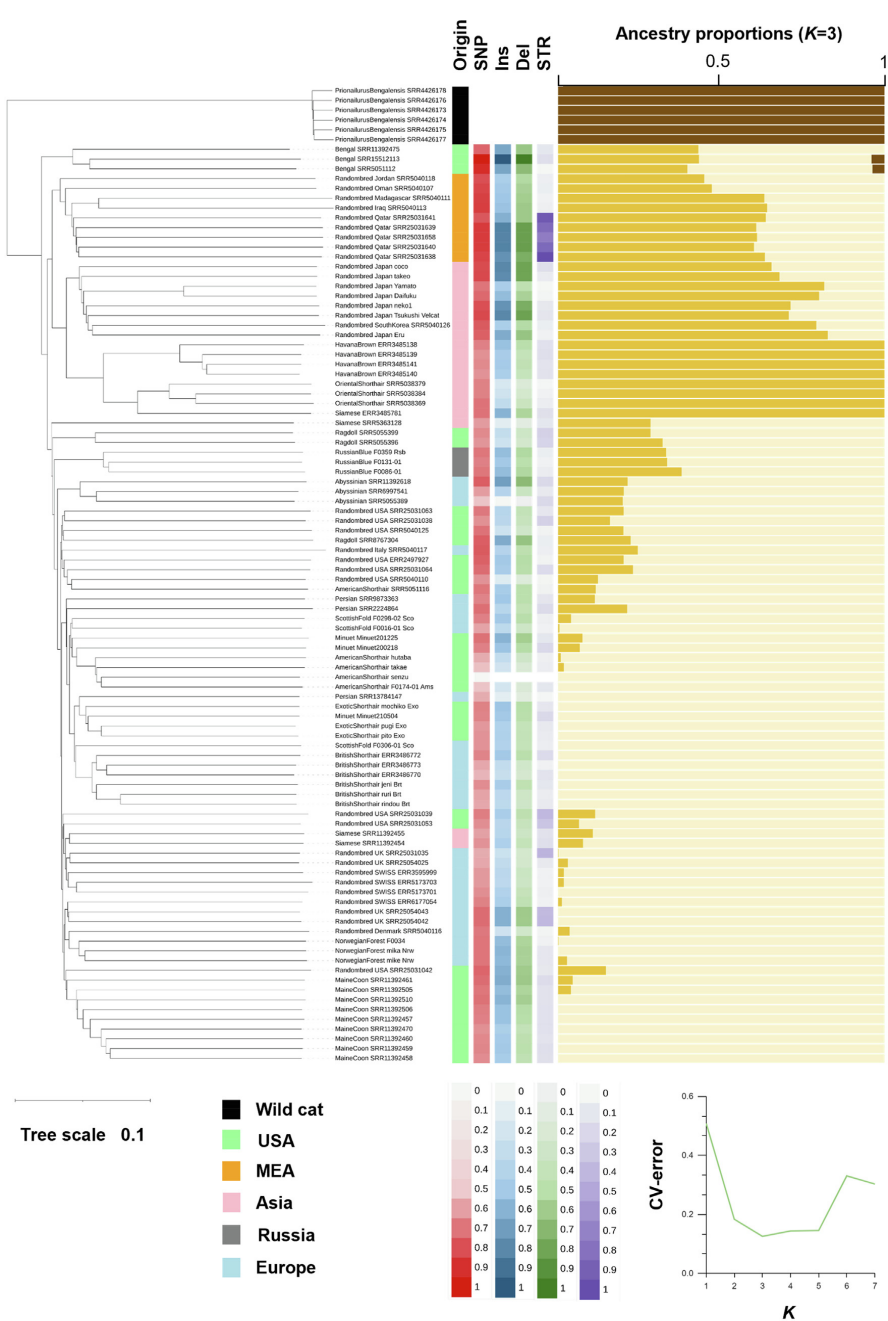
Population genomics for 100 feline WGS data based on AnAms1.0 genome assembly. Phylogenetic analyses were conducted based on neighbor-joining method. Gray circle indicates bootstrap support of > 95. The origin was depicted in each color based on the original geographic regions or the place where the breed developed or the origin of the ancestorial breed. The number of SNP, insertion (Ins), deletions (Del) and structural variations (STR) were normalized. The right bar graph indicates ADMIXTURE results based on the lowest cross-validation errors (*K* = 3).

The genetic characterization was conducted by genome-wide SNP data from 15 inbred, four random-bred and a wild cat population. Random-bred cats from Africa had a larger number of Insertions, Deletions and Structural variants compared to other groups, which indicates the ancestry variants (Fig. 4).

The phylogenetic analysis based on the Neighbor joining method indicated four major groups including the Asian group, the European/American group, the Bengal Cat, and the wild cat (*Prionailurus bengalensis*). Cross-validation error of ADMIXTURE analyses indicates three ancestries is plausible and it indicates three genetic ancestry, wild cat, Asian, and European/American group (Fig. 4).

### Database construction

We created Cats-I, a genome database for AnAms1.0, comprising a genome browser with annotated genes, expression profiles, BLAST search tools, and download tools for genomic resources (available at https://cat.annotation.jp).

## Discussion

In this study, we constructed AnAms1.0, a chromosome-scale assembly of the American Shorthair breed, improving on existing domestic cat assemblies. Considering the lowest number of scaffolds, the longest N50 length, the highest QV, the lowest error rate and the best score in complete and missing BUSCOs, AnAms1.0 is a high-quality Felidae genome assembly. Additionally, AnAms1.0 has a larger number of SVs and intact OR genes than the widely used felCat9 assembly. A total of 1,685 novel protein-coding genes were annotated based on our assembly. Collectively, this genome assembly and its novel annotations hold the potential to detect small variations and large SVs in Felidae.

### Accuracy of mapping and variant detection

The mapping rate in AnAms1.0 was higher than that in felCat9 for 100 cats from various origins. Collectively, AnAms1.0 is more informative than felCat9 when the Illumina short-read data for the domestic and leopard cat genomes are used.

Our short-read-based analyses uncovered a difference in small variant numbers. A genome-wide population genetic analyses indicate that both Abyssinians and American Shorthairs share origins with US and European populations, resulting in relatively small genetic differences between the two breeds. Notably, a one-base deletion, identified as a false positive through validation with Sanger sequencing, was found in felCat9 but not detected in the AnAms1.0 assembly [69]. Consequently, the observed results may be primarily influenced by mis-assembly in the felCat9 genome.

### Insights for feline precision medicine

Recent advancements in research have moved toward precision medicine in companion animals [19], [70]. A recent genomic study on muscular dystrophy in cats found that the AnAms 1.0 assembly is of higher quality than the felCat9 assembly [69]. This study identified a false positive 1 bp deletion in the *DMD* gene, which is responsible for muscular dystrophy, in felCat9 but not in AnAms 1.0. This discrepancy indicates a mis-assembly in the felCat9 genome. Small genetic variants like this can significantly impact genetic traits and diseases in domestic cats [71], underscoring the importance of accurate small variant detection in precision medicine. Therefore, our data, which includes the AnAms 1.0 assembly and its annotations, provides a more reliable option for detecting variants with high accuracy, although future assessments of other cases of mis-assembly are necessary.

We identified over 1.5 thousand SVs and 29 million repetitive regions in AnAms1.0 that were not present in the previous domestic cat assembly. Our *in silico* analyses using DAVID and IPA identified cardiomyopathy-related genes enriched in the identified SV candidates.

Cardiomyopathy is a significant genetic disorder in domestic cats, with mutations that likely contribute to predisposition and pathogenesis remaining unidentified [72]. In addition SVs, the presence of repetitive sequences has also been linked to genetic diseases [73], [74]. Given that both factors are implicated in various genetic disorders [2], [75], the AnAms1.0 assembly is anticipated to offer valuable insights for precision medicine in domestic cats and aid in the characterization of specific traits. In this study, we compared the AnAms1.0 and felCat9 assemblies and identified variant differences that may be attributed to breed and/or individual variations. Since long-read sequencers are effective in detecting SVs and repetitive sequences in humans [73], [76], further genomic analyses of SVs across more breeds and individuals are necessary to advance the pan-genome analysis in cats.

### Limitation for mitochondrial DNA and Y chromosome

AnAms1.0 was assembled from the genome of a female cat; therefore the Y chromosome sequence was not obtained. The mitochondrial genome sequence was also not determined as mitochondrial DNA was eliminated before reading sequences to facilitate accurate nuclear genome extraction. Therefore, further studies focused on the sequencing and characterization of the male cat genome are required to determine the Y chromosome and mitochondrial DNA sequences.

## Conclusion

The robust genome assembly proposed in our study, coupled with novel annotations, not only advances feline genomic research but also holds promise for enhancing precision veterinary medicine. The identified genetic variations, especially in disease-related genes, highlight the potential for informed clinical decision-making and policy development in feline healthcare, laying a foundation for targeted interventions and breed-specific healthcare strategies.

## Compliance with ethics requirements

All Institutional and National Guidelines for the care and use of animals were followed.

## Declaration of competing interest

The authors declare that they have no known competing financial interests or personal relationships that could have appeared to influence the work reported in this paper.

## References

[1] Field M.A., Rosen B.D., Dudchenko O., Chan E.K.F., Minoche A.E., Edwards R.J. (2020). Canfam_GSD: De novo chromosome-length genome assembly of the German Shepherd Dog (Canis lupus familiaris) using a combination of long reads, optical mapping, and Hi-C. GigaScience.

[2] Buckley R.M., Davis B.W., Brashear W.A., Farias F.H.G., Kuroki K., Graves T. (2020). A new domestic cat genome assembly based on long sequence reads empowers feline genomic medicine and identifies a novel gene for dwarfism. PLoS Genet.

[3] Parker H.G., Dreger D.L., Rimbault M., Davis B.W., Mullen A.B., Carpintero-Ramirez G. (2017). Genomic Analyses Reveal the Influence of Geographic Origin, Migration, and Hybridization on Modern Dog Breed Development. Cell Rep.

[4] Morrill K., Hekman J., Li X., McClure J., Logan B., Goodman L. (2022). Ancestry-inclusive dog genomics challenges popular breed stereotypes. Science.

[5] Buckley R.M., Lyons L.A. (2020). Precision/Genomic Medicine for Domestic Cats. Vet Clin North Am Small Anim Pract.

[6] Driscoll C.A., Clutton-Brock J., Kitchener A.C., O’Brien S.J. (2009). The Taming of the cat. Genetic and archaeological findings hint that wildcats became housecats earlier–and in a different place–than previously thought. Sci Am.

[7] Dennis-Bryan K. (2013). The Complete Cat Breed Book. DK Publishing.

[8] Bell J, Cavanagh K, Tilley LP, Smith FWK. Veterinary medical guide to dog and cat breeds. Jackson, MS: Teton NewMedia; 2012. doi: 10.1201/b16185.

[9] Pontius JU, Mullikin JC, Smith DR, Agencourt Sequencing Team, Lindblad-Toh K, Gnerre S, et al. Initial sequence and comparative analysis of the cat genome. Genome Res 2007;17:1675–89. 10.1101/gr.6380007 PMC2045150 17975172

[10] Lipinski M.J., Amigues Y., Blasi M., Broad T.E., Cherbonnel C., Cho G.J. (2007). An international parentage and identification panel for the domestic cat (Felis catus). Anim Genet.

[11] Menotti-Raymond M., David V.A., Pflueger S.M., Lindblad-Toh K., Wade C.M., O’Brien S.J. (2008). Patterns of molecular genetic variation among cat breeds. Genomics.

[12] Kurushima J.D., Lipinski M.J., Gandolfi B., Froenicke L., Grahn J.C., Grahn R.A. (2013). Variation of cats under domestication: genetic assignment of domestic cats to breeds and worldwide random-bred populations. Anim Genet.

[13] Gandolfi B., Alhaddad H., Abdi M., Bach L.H., Creighton E.K., Davis B.W. (2018). Applications and efficiencies of the first cat 63K DNA array. Sci Rep.

[14] Matsumoto Y., Ruamrungsri N., Arahori M., Ukawa H., Ohashi K., Lyons L.A. (2021). Genetic relationships and inbreeding levels among geographically distant populations of Felis catus from Japan and the United States. Genomics.

[15] Aberdein D., Munday J.S., Gandolfi B., Dittmer K.E., Malik R., Garrick D.J. (2017). A FAS-ligand variant associated with autoimmune lymphoproliferative syndrome in cats. Mamm Genome.

[16] Lyons L.A., Creighton E.K., Alhaddad H., Beale H.C., Grahn R.A., Rah H. (2016). Whole genome sequencing in cats, identifies new models for blindness in AIPL1 and somite segmentation in HES7. BMC Genomics.

[17] Xu X., Sun X., Hu X.-S., Zhuang Y., Liu Y.-C., Meng H. (2016). Whole Genome Sequencing Identifies a Missense Mutation in HES7 Associated with Short Tails in Asian Domestic Cats. Sci Rep.

[18] Bertolini F., Gandolfi B., Kim E.S., Haase B., Lyons L.A., Rothschild M.F. (2016). Evidence of selection signatures that shape the Persian cat breed. Mamm Genome.

[19] Mauler D.A., Gandolfi B., Reinero C.R., O’Brien D.P., Spooner J.L., Lyons L.A. (2017). Precision medicine in cats: Novel Niemann-pick type C1 diagnosed by whole-genome sequencing. J Vet Intern Med.

[20] O’Brien S.J., Johnson W., Driscoll C., Pontius J., Pecon-Slattery J., Menotti-Raymond M. (2008). State of cat genomics. Trends Genet.

[21] de Jong T.V., Chen H., Brashear W.A., Kochan K.J., Hillhouse A.E., Zhu Y. (2022). mRatBN7.2: familiar and unfamiliar features of a new rat genome reference assembly. Physiol Genomics.

[22] Cat Fanciers’ Association. CFA Breed Standards. 2022.

[23] Chin C.-S., Peluso P., Sedlazeck F.J., Nattestad M., Concepcion G.T., Clum A. (2016). Phased diploid genome assembly with single-molecule real-time sequencing. Nat Methods.

[24] Walker B.J., Abeel T., Shea T., Priest M., Abouelliel A., Sakthikumar S. (2014). Pilon: an integrated tool for comprehensive microbial variant detection and genome assembly improvement. PLoS One.

[25] Marçais G., Kingsford C. (2011). A fast, lock-free approach for efficient parallel counting of occurrences of k-mers. Bioinformatics.

[26] Putnam N.H., O’Connell B.L., Stites J.C., Rice B.J., Blanchette M., Calef R. (2016). Chromosome-scale shotgun assembly using an in vitro method for long-range linkage. Genome Res.

[27] Alonge M., Soyk S., Ramakrishnan S., Wang X., Goodwin S., Sedlazeck F.J. (2019). RaGOO: fast and accurate reference-guided scaffolding of draft genomes. Genome Biol.

[28] Simão F.A., Waterhouse R.M., Ioannidis P., Kriventseva E.V., Zdobnov E.M. (2015). BUSCO: assessing genome assembly and annotation completeness with single-copy orthologs. Bioinformatics.

[29] Kurtz S., Phillippy A., Delcher A.L., Smoot M., Shumway M., Antonescu C. (2004). Versatile and open software for comparing large genomes. Genome Biol.

[30] Rhie A., Walenz B.P., Koren S., Phillippy A.M. (2020). Merqury: reference-free quality, completeness, and phasing assessment for genome assemblies. Genome Biol.

[31] Li H. (2018). Minimap2: pairwise alignment for nucleotide sequences. Bioinformatics.

[32] Wu T.D., Watanabe C.K. (2005). GMAP: a genomic mapping and alignment program for mRNA and EST sequences. Bioinformatics.

[33] Hoff K.J., Lange S., Lomsadze A., Borodovsky M., Stanke M. (2016). BRAKER1: Unsupervised RNA-Seq-Based Genome Annotation with GeneMark-ET and AUGUSTUS. Bioinformatics.

[34] Stanke M., Schöffmann O., Morgenstern B., Waack S. (2006). Gene prediction in eukaryotes with a generalized hidden Markov model that uses hints from external sources. BMC Bioinf.

[35] Stanke M., Diekhans M., Baertsch R., Haussler D. (2008). Using native and syntenically mapped cDNA alignments to improve de novo gene finding. Bioinformatics.

[36] Cantarel B.L., Korf I., Robb S.M.C., Parra G., Ross E., Moore B. (2008). MAKER: an easy-to-use annotation pipeline designed for emerging model organism genomes. Genome Res.

[37] Shumate A., Salzberg S.L. (2021). Liftoff: accurate mapping of gene annotations. Bioinformatics.

[38] Dunn N.A., Unni D.R., Diesh C., Munoz-Torres M., Harris N.L., Yao E. (2019). Apollo: Democratizing genome annotation. PLoS Comput Biol.

[39] Pertea G., Pertea M. (2020). GFF Utilities: GffRead and GffCompare. F1000Res.

[40] Jones P., Binns D., Chang H.-Y., Fraser M., Li W., McAnulla C. (2014). InterProScan 5: genome-scale protein function classification. Bioinformatics.

[41] Moriya Y., Itoh M., Okuda S., Yoshizawa A.C., Kanehisa M. (2007). KAAS: an automatic genome annotation and pathway reconstruction server. Nucleic Acids Res.

[42] Marchler-Bauer A., Bryant S.H. (2004). CD-Search: protein domain annotations on the fly. Nucleic Acids Res.

[43] Shen W., Le S., Li Y., Hu F. (2016). SeqKit: A Cross-Platform and Ultrafast Toolkit for FASTA/Q File Manipulation. PLoS One.

[44] Niimura Y. (2013). Identification of olfactory receptor genes from mammalian genome sequences. Methods Mol Biol.

[45] Niimura Y., Matsui A., Touhara K. (2018). Acceleration of Olfactory Receptor Gene Loss in Primate Evolution: Possible Link to Anatomical Change in Sensory Systems and Dietary Transition. Mol Biol Evol.

[46] Langmead B., Salzberg S.L. (2012). Fast gapped-read alignment with Bowtie 2. Nat Methods.

[47] Li H., Handsaker B., Wysoker A., Fennell T., Ruan J., Homer N. (2009). The Sequence Alignment/Map format and SAMtools. Bioinformatics.

[48] Bolger A.M., Lohse M., Usadel B. (2014). Trimmomatic: a flexible trimmer for Illumina sequence data. Bioinformatics.

[49] Kim D., Langmead B., Salzberg S.L. (2015). HISAT: a fast spliced aligner with low memory requirements. Nat Methods.

[50] Kovaka S., Zimin A.V., Pertea G.M., Razaghi R., Salzberg S.L., Pertea M. (2019). Transcriptome assembly from long-read RNA-Seq alignments with StringTie2. Genome Biol.

[51] Grabherr M.G., Haas B.J., Yassour M., Levin J.Z., Thompson D.A., Amit I. (2011). Full-length transcriptome assembly from RNA-Seq data without a reference genome. Nat Biotechnol.

[52] Nawrocki E.P., Eddy S.R. (2013). Infernal 1.1: 100-fold faster RNA homology searches. Bioinformatics.

[53] Kalvari I., Nawrocki E.P., Ontiveros-Palacios N., Argasinska J., Lamkiewicz K., Marz M. (2021). Rfam 14: expanded coverage of metagenomic, viral and microRNA families. Nucleic Acids Res.

[54] Wang L., Park H.J., Dasari S., Wang S., Kocher J.-P., Li W. (2013). CPAT: Coding-Potential Assessment Tool using an alignment-free logistic regression model. Nucleic Acids Res.

[55] Altschul S.F., Gish W., Miller W., Myers E.W., Lipman D.J. (1990). Basic local alignment search tool. J Mol Biol.

[56] Sievers F., Wilm A., Dineen D., Gibson T.J., Karplus K., Li W. (2011). Fast, scalable generation of high-quality protein multiple sequence alignments using Clustal Omega. Mol Syst Biol.

[57] Waterhouse A.M., Procter J.B., Martin D.M.A., Clamp M., Barton G.J. (2009). Jalview Version 2–a multiple sequence alignment editor and analysis workbench. Bioinformatics.

[58] Frankish A., Diekhans M., Jungreis I., Lagarde J., Loveland J.E., Mudge J.M. (2021). GENCODE 2021. Nucleic Acids Res.

[59] Quinlan A.R., Hall I.M. (2010). BEDTools: a flexible suite of utilities for comparing genomic features. Bioinformatics.

[60] Yuan Y., Bayer P.E., Lee H.-T., Edwards D. (2017). runBNG: a software package for BioNano genomic analysis on the command line. Bioinformatics.

[61] Wang M., Kong L. (2019). pblat: a multithread blat algorithm speeding up aligning sequences to genomes. BMC Bioinf.

[62] Zhao H., Sun Z., Wang J., Huang H., Kocher J.-P., Wang L. (2014). CrossMap: a versatile tool for coordinate conversion between genome assemblies. Bioinformatics.

[63] Huang D.W., Sherman B.T., Tan Q., Collins J.R., Alvord W.G., Roayaei J. (2007). The DAVID Gene Functional Classification Tool: a novel biological module-centric algorithm to functionally analyze large gene lists. Genome Biol.

[64] Krämer A., Green J., Pollard J., Tugendreich S. (2014). Causal analysis approaches in Ingenuity Pathway Analysis. Bioinformatics.

[65] Chang C.C., Chow C.C., Tellier L.C., Vattikuti S., Purcell S.M., Lee J.J. (2015). Second-generation PLINK: rising to the challenge of larger and richer datasets. GigaScience.

[66] Alexander D.H., Novembre J., Lange K. (2009). Fast model-based estimation of ancestry in unrelated individuals. Genome Res.

[67] Huson D.H., Bryant D. (2024). The SplitsTree App: interactive analysis and visualization using phylogenetic trees and networks. Nat Methods.

[68] Niimura Y. (2012). Olfactory receptor multigene family in vertebrates: from the viewpoint of evolutionary genomics. Curr Genomics.

[69] Yokoyama N., Matsumoto Y., Yamaguchi T., Okada K., Kinoshita R., Shimbo G. (2024). A de novo nonsense variant in the DMD gene associated with X-linked dystrophin-deficient muscular dystrophy in a cat. J Vet Intern Med.

[70] Mealey K.L., Martinez S.E., Villarino N.F., Court M.H. (2019). Personalized medicine: going to the dogs?. Hum Genet.

[71] Lyons L.A. (2015). DNA mutations of the cat: the good, the bad and the ugly. J Feline Med Surg.

[72] Kittleson M.D., Meurs K.M., Harris S.P. (2015). The genetic basis of hypertrophic cardiomyopathy in cats and humans. J Vet Cardiol.

[73] Sone J., Mitsuhashi S., Fujita A., Mizuguchi T., Hamanaka K., Mori K. (2019). Long-read sequencing identifies GGC repeat expansions in NOTCH2NLC associated with neuronal intranuclear inclusion disease. Nat Genet.

[74] Malik I., Kelley C.P., Wang E.T., Todd P.K. (2021). Molecular mechanisms underlying nucleotide repeat expansion disorders. Nat Rev Mol Cell Biol.

[75] Olsson M., Meadows J.R.S., Truvé K., Rosengren Pielberg G., Puppo F., Mauceli E. (2011). A novel unstable duplication upstream of HAS2 predisposes to a breed-defining skin phenotype and a periodic fever syndrome in Chinese Shar-Pei dogs. PLoS Genet.

[76] Logsdon G.A., Vollger M.R., Eichler E.E. (2020). Long-read human genome sequencing and its applications. Nat Rev Genet.

