# Supplementary material for: Chromosome-scale assembly with improved annotation provides insights into breed-wide genomic structure and diversity in domestic cats
Source: J Adv Res. 2024 Oct 28;75:863–74. doi: 10.1016/j.jare.2024.10.023 (PMC12789764; doi:10.1016/j.jare.2024.10.023)
Supplement: Supplementary Data 2 [file mmc2.docx]

# Supporting information

**lncRNA analyses**

lncRNA conservation was evaluated via alignment to the human reference genome and leveraging the conservation scores, including phastCons and phyloP, obtained from the 100-way conservation track. The four classes of lncRNA genes were generally less conserved in terms of sequence homology to human genes, whereas the protein-coding sequences were conserved across 100 mammalian species (Fig. S11B). Nonetheless, a few cat lncRNA genes were highly conserved with reference to the human genome, such as TGFB2-OT1 and AC004540.2 (Fig. S11C), the functions of which remain unknown.

To further infer the functions of lncRNA genes, we performed functional annotation using the protein-coding counterparts of the antisense and intronic lncRNA genes. The top annotated Gene Ontology terms suggested that hundreds of these lncRNA genes could be related to transcription and signaling regulation. In contrast, the top annotated KEGG pathways linked these genes to various disease conditions and signaling pathways (Fig. S11D-E).

## Supplementary tables

**Table S1.** Olfactory receptor (OR) genes in AnAms1.0 and the corresponding genes in felCat9.

**Table S2.** Whole-genome sequencing data used for variant detection.

**Table S3.** Status of genome assembly with PacBio, Illumina PE, and Hi-C reads.

**Table S4.** Table S4. Lengths and number of 'Post-OM hybrid scaffolds' consisting of chromosome-level scaffolds of AnAms1.0.

**Table S5.** Length and ratio of repetitive sequences.

**Table S6.** Statistics of *t*-tests for variant calling between references.

**Table S7.** Number of structural variants in the Senzu genome against felCat9.

**Table S8.** Statistics of Iso-Seq assembly from the ovary, oviduct, and uterine tissues of Senzu and Takae.

**Table S9.**Curated levels of protein-coding loci.

**Table S10.** Comparison of the number of protein-coding loci with that in felCat9.

**Table S11.** Correspondence between regions of significant gene duplication in AnAms1.0 and felCat9**.**

**Table S12.** Number of olfactory receptor genes in cat genome assemblies.

## Supplementary figures

**Fig. S1.** Overview of the protein-coding gene structural annotation.

**Fig. S2.** Overview of lncRNA prioritization using an integrated method.

**Fig. S3.** Length distribution of optical mapping data. (A) Length distribution of raw molecules 20 Kbp to 1 Mbp in length. (B) Length distribution of assembled genome maps.

**Fig. S4.** Results of optical mapping and hybrid scaffolding. Scaffolding 87 of 372 input Hi-C sequences (99.4% length) based on OM data resulted in 37 super-scaffolds.

**Fig. S5.** Comparison of the number of single-nucleotide polymorphisms (SNPs), insertions, and deletions between AnAms 1.0 and felCat9. (A) SNPs, (B) insertions, (C) deletions.

**Fig. S6.** Distribution of mapped Senzu PacBio read depth on the felCat9 genome. The X- and Y-axes indicate physical position (Mb) and mapped read depth (×), respectively. The scale of E3 is different from that in other graphs to show the chromosomal deletion.

**Fig. S7.** SV size and location distribution. Size and location distribution plot of insertions and deletions detected using optical mapping data from the American Shorthair cat against the felCat9 genome sequence assembly from the Abyssinian cat. Positive and negative sizes indicate insertions and deletions in AnAms1.0 relative to felCat9, respectively. SV, structural variation.

**Fig. S8.** Functional enrichment analysis via DAVID. Top five enriched Gene Ontology (GO) or Kyoto Encyclopedia of Genes and Genomes (KEGG) pathway terms in the combined insertion/deletion (INDEL), insertion (INS), or deletion (DEL) categories. The X-axis denotes the affected gene counts. Bars are ranked in ascending order of p-values from top to bottom in each subplot. Terms related to cardiac function are highlighted in red.

**Fig. S9.** Variant analysis by IPA. Top 20 enriched canonical pathways affected by genome-wide deletions in AnAms1.0 with respect to felCat9. The vertical line at 1.3 indicates the significance threshold of p-value < 0.05 (Fisher's Exact Test). Most results were related to cardiac function, highlighted in red, as well as neuronal signaling and development. Specifically, enhanced cardiac hypertrophy was predicted, with sensitivity to the vasodilating drug Sildenafil.

**Fig. S10. Functional annotation of protein-coding genes and lncRNAs.** (A) Summary of functional annotation. The top row shows the number of genes in each eukaryotic ortholog group (KOG) and the top 15 with the highest number. The middle and bottom rows show the top 15 Kyoto Encyclopedia of Genes and Genomes (KEGG) terms and the top 15 annotations of Pfam, respectively. The right side of the bar shows the description of the annotation, and the white numbers inside the bar indicate the number of genes. (B–C) The distribution of conservation scores, average phastCons scores, and average phyloP scores is summarized for the four classes of lncRNAs and protein-coding transcripts. Total number of transcripts and the percentage of transcripts mapped to the human reference genome are shown. (C) Two lncRNAs conserved between cat and human are shown in wrapped multiple tracks. The three tracks show nucleotide sequences of cat transcripts, the human counterpart, and the level of sequence consensus, respectively. (D–E) Functional annotation. Numbers of protein-coding genes belonging to the top five Gene Ontology terms (including biological process [BP], cellular component [CC], and molecular function [MF] categories) and the top ten KEGG pathway terms are summarized for the associated (D) antisense and (E) intronic lncRNA loci.

**Fig. S11.** Comparison of Chromosome D2 between AnAms 1.0 and felCat9.

**Fig. S12.** Comparison of the genomic regions containing OR gene clusters between AnAms1.0 and felCat9. A long/short vertical bar in blue/red is explained in the legend of Fig 4. A green vertical bar indicates an OR gene identified from AnAms1.0 with a corresponding OR gene (with a >97% amino acid sequence identity) in the felCat9 sequence. However, the felCat9 gene is encoded in a short contig (e.g., chrB2_random_ctg521) that is not assembled into a chromosome. The scale bar indicates physical distance.

**Fig. S13.** Read-based assessment around the OR genes on chromosome D2. WGS data from five American shorthairs and three Abyssinians were mapped to AnAms1.0 and felCat9 using DRAGEN to obtain bam files. The region of chromosome D2 was extracted from bam files using samtools(1), and bigwig files were created using deeptools (v3.5.5., (2)) from bam files. Plots of the regions between ARHGAP22 and TMEM72, which contained OR genes, were drawn by pyGenomeTracks (v3.9, (3,4)).

**Supporting data**

AGP file and Appendix for functional annotation are provided on Google Drive (https://drive.google.com/drive/folders/13G0G5ajURhEfsFPBhY-dMnVoBRFLmAg5?usp=sharing)

**References**

(1) Danecek P, Bonfield JK, Liddle J, Marshall J, Ohan V, Pollard MO, Whitwham A, Keane T, McCarthy SA, Davies RM, Li H, Twelve years of SAMtools and BCFtools, GigaScience (2021) 10(2) giab008 [33590861]

(2) Ramírez, Fidel, Devon P. Ryan, Björn Grüning, Vivek Bhardwaj, Fabian Kilpert, Andreas S. Richter, Steffen Heyne, Friederike Dündar, and Thomas Manke. deepTools2: A next Generation Web Server for Deep-Sequencing Data Analysis. Nucleic Acids Research (2016). doi:10.1093/nar/gkw257.

(3) Fidel Ramírez, Vivek Bhardwaj, Laura Arrigoni, Kin Chung Lam, Björn A. Grüning, José Villaveces, Bianca Habermann, Asifa Akhtar & Thomas Manke. High-resolution TADs reveal DNA sequences underlying genome organization in flies. Nature Communications (2018) doi:10.1038/s41467-017-02525-w.

(4) Lopez-Delisle L, Rabbani L, Wolff J, Bhardwaj V, Backofen R, Grüning B, Ramírez F, Manke T. pyGenomeTracks: reproducible plots for multivariate genomic data sets. Bioinformatics. 2020 Aug 3:btaa692. doi: 10.1093/bioinformatics/btaa692. Epub ahead of print. PMID: 32745185.
