# Supplementary material for: Chromosome-scale assembly with improved annotation provides insights into breed-wide genomic structure and diversity in domestic cats
Source: J Adv Res. 2024 Oct 28;75:863–74. doi: 10.1016/j.jare.2024.10.023 (PMC12789764; doi:10.1016/j.jare.2024.10.023)
Supplement: Supplementary Data 3 [file mmc3.pptx]

## Slide 1
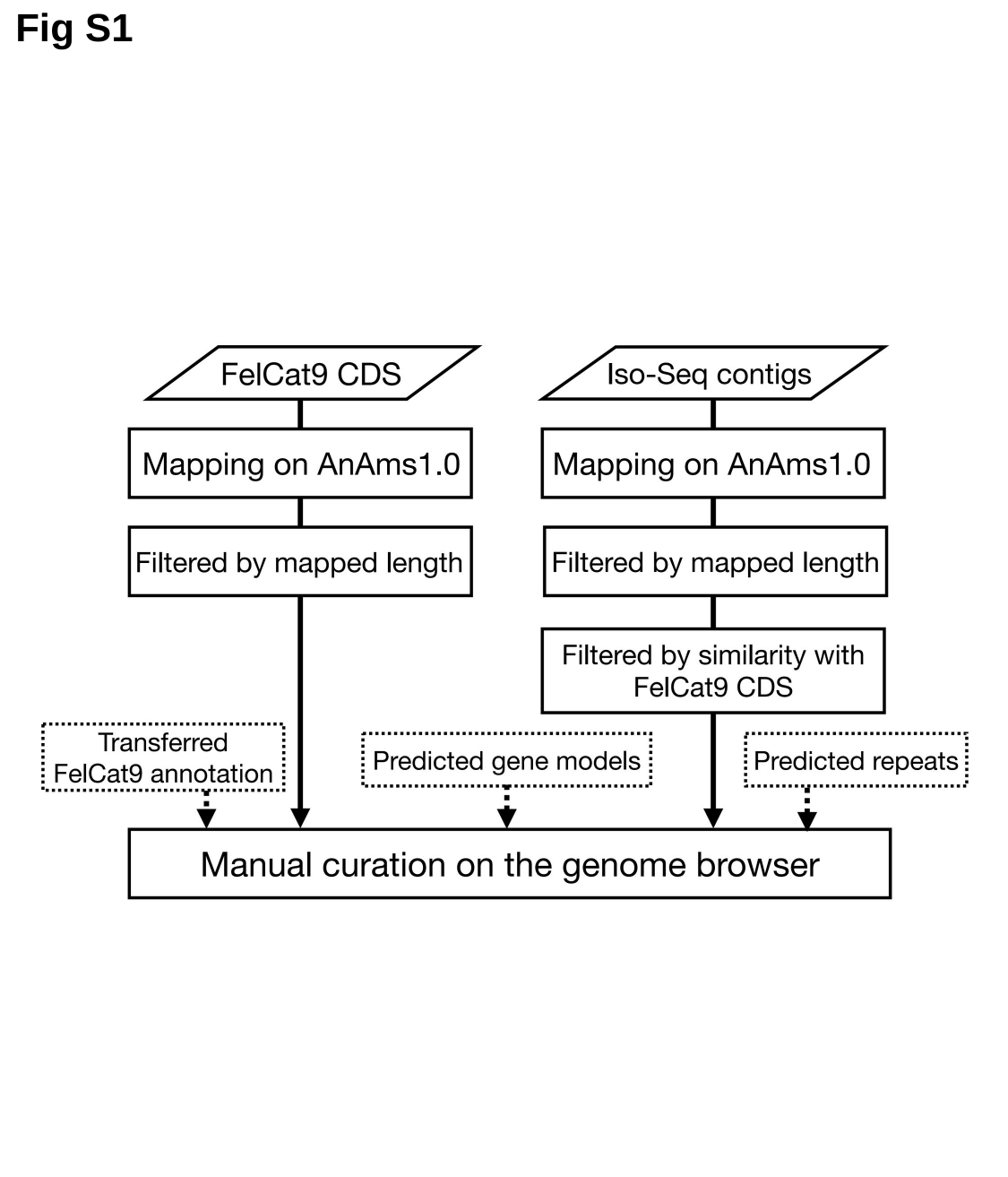

Fig S1

## Slide 2
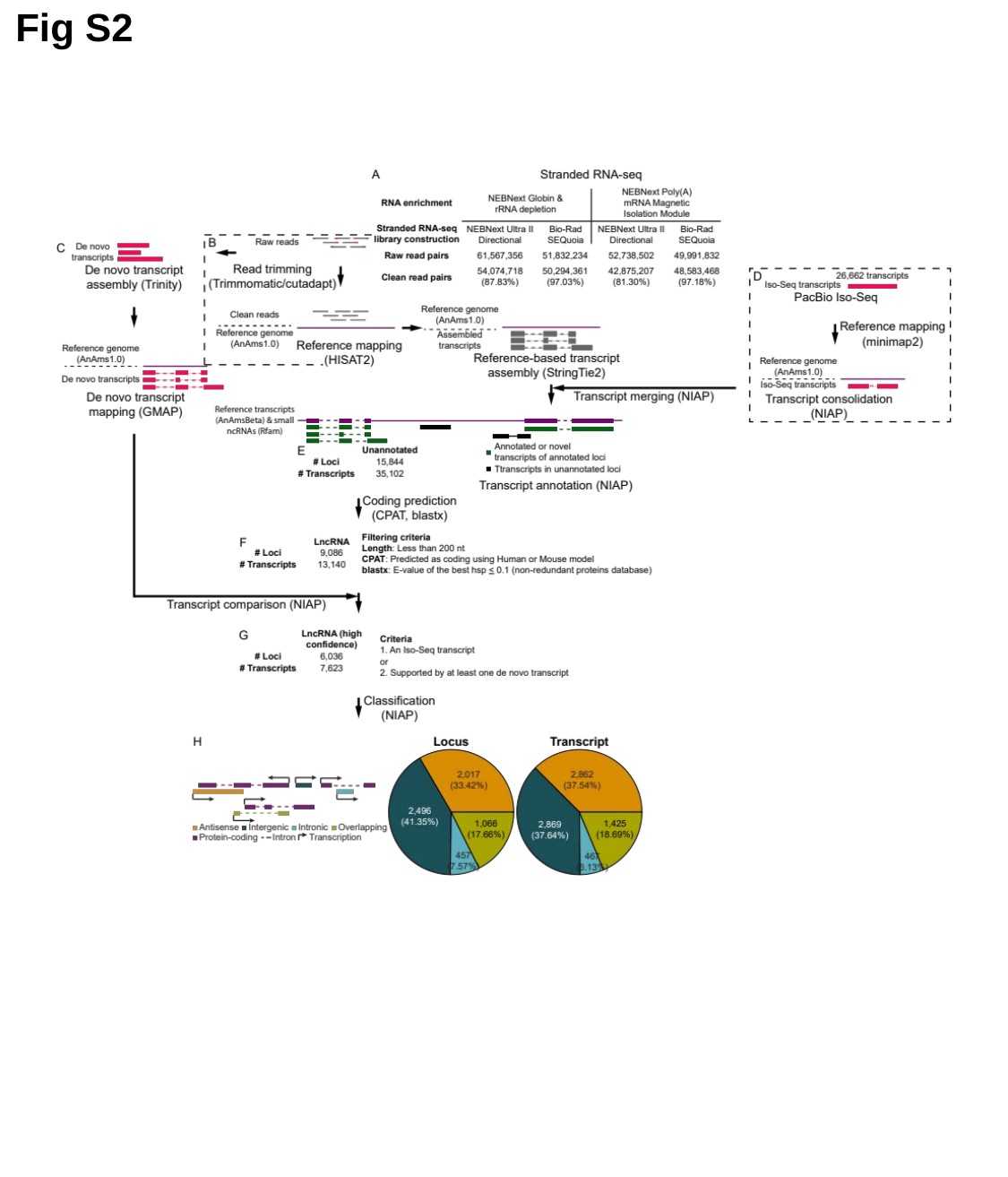

Fig S2

## Slide 3
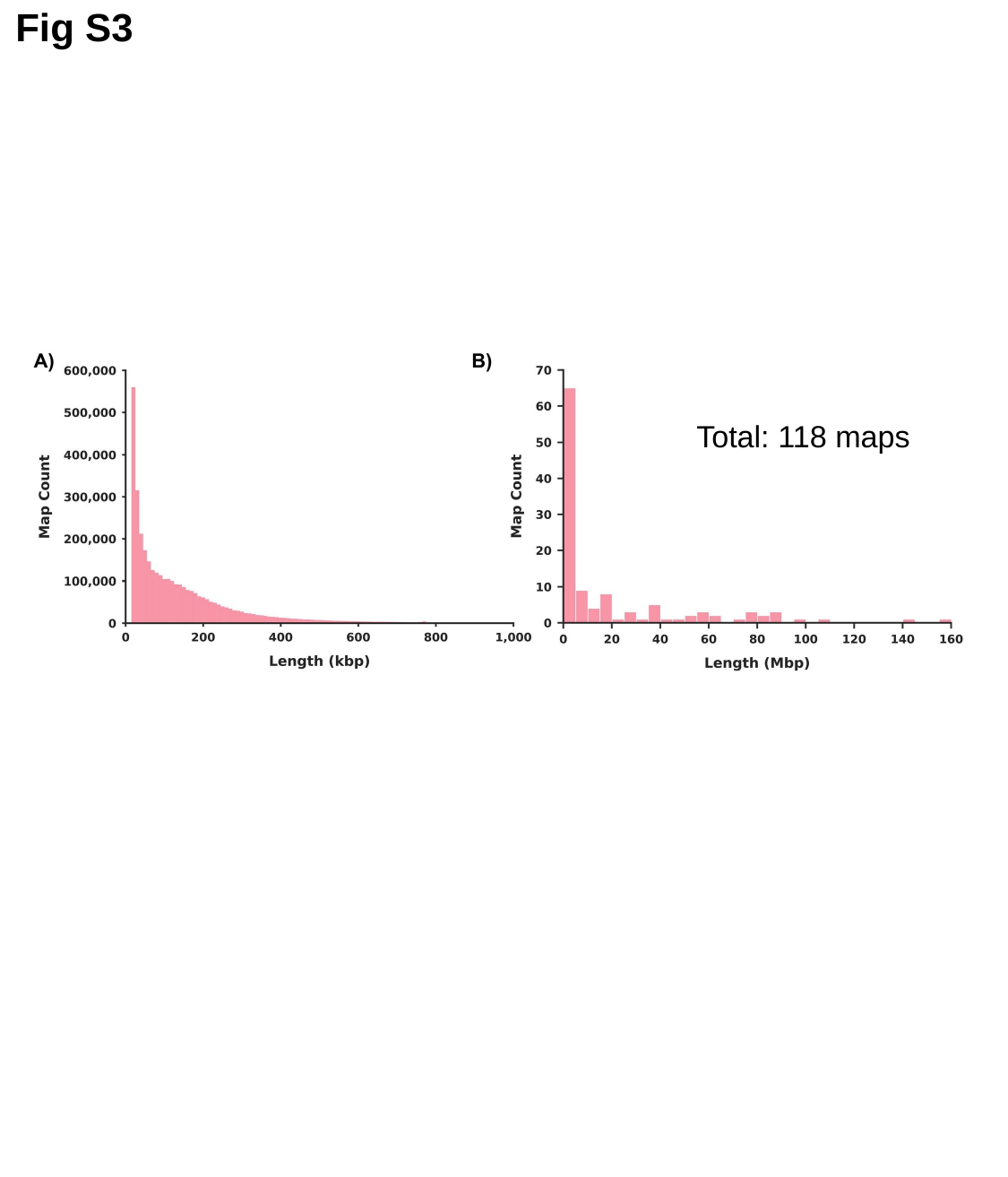

Fig S3
Total: 118 maps

## Slide 4
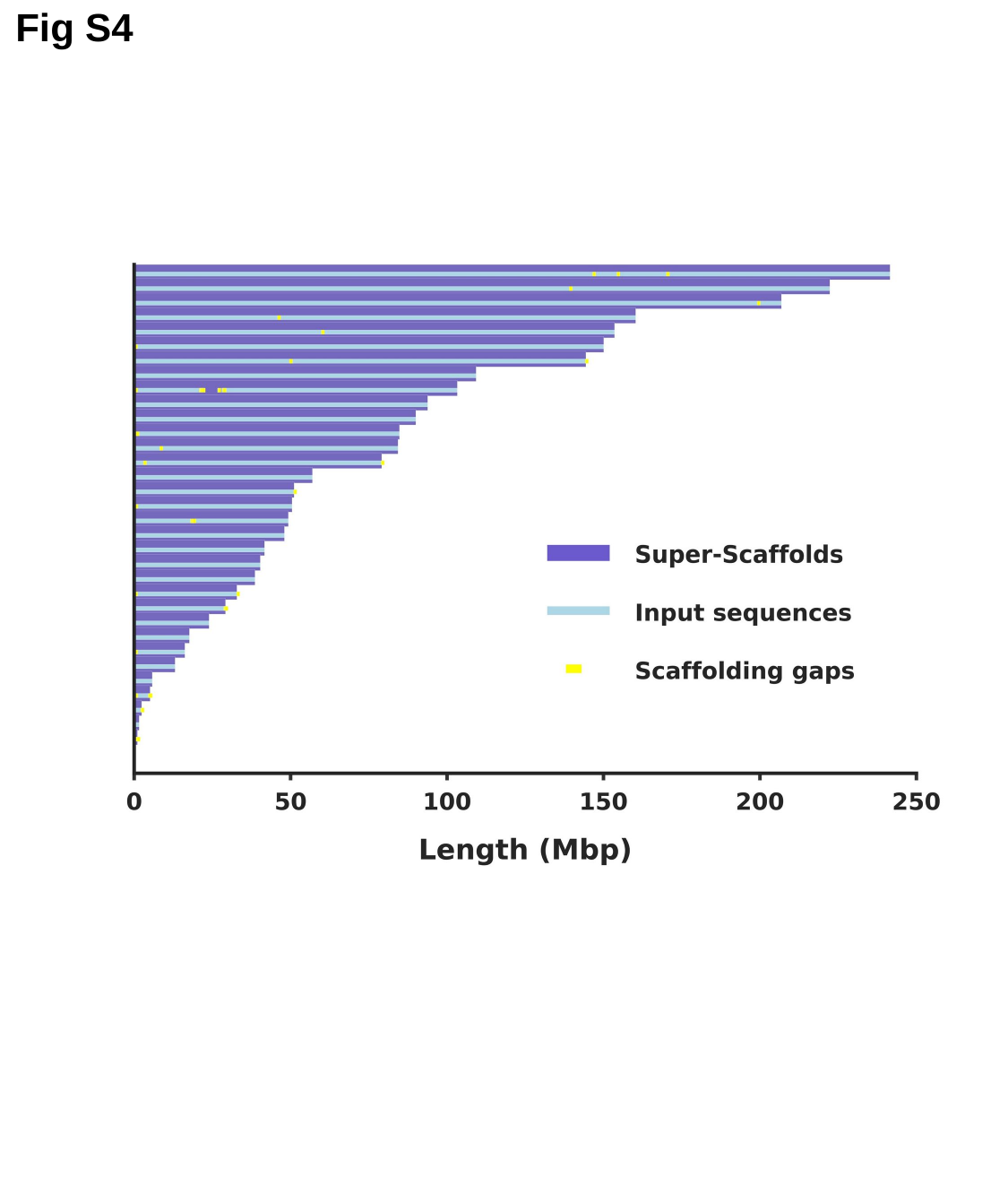

Fig S4

## Slide 5
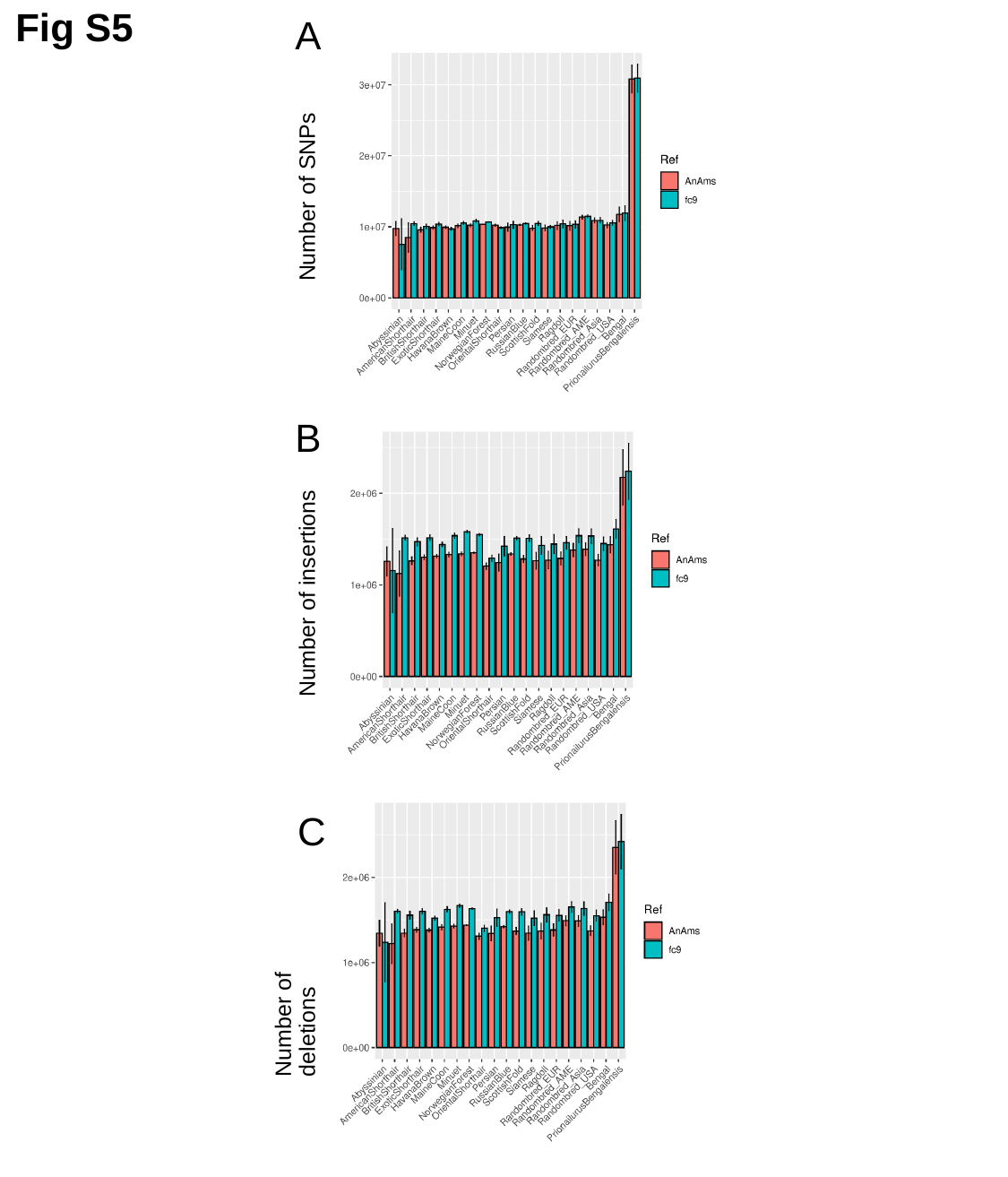

Fig S5
A
Number of SNPs
B
Number of insertions
C
Number of deletions

## Slide 6
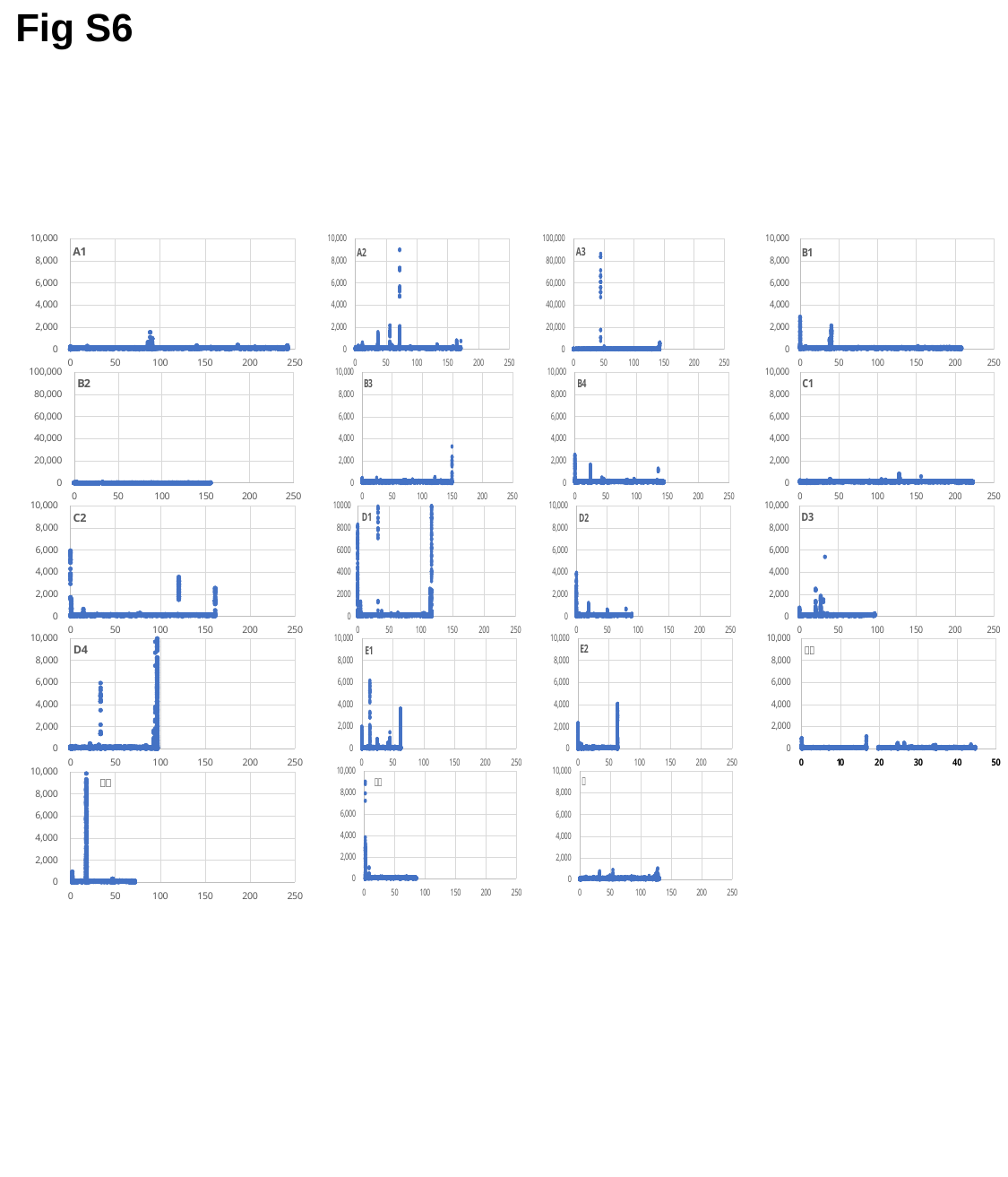

Fig S6

## Slide 7
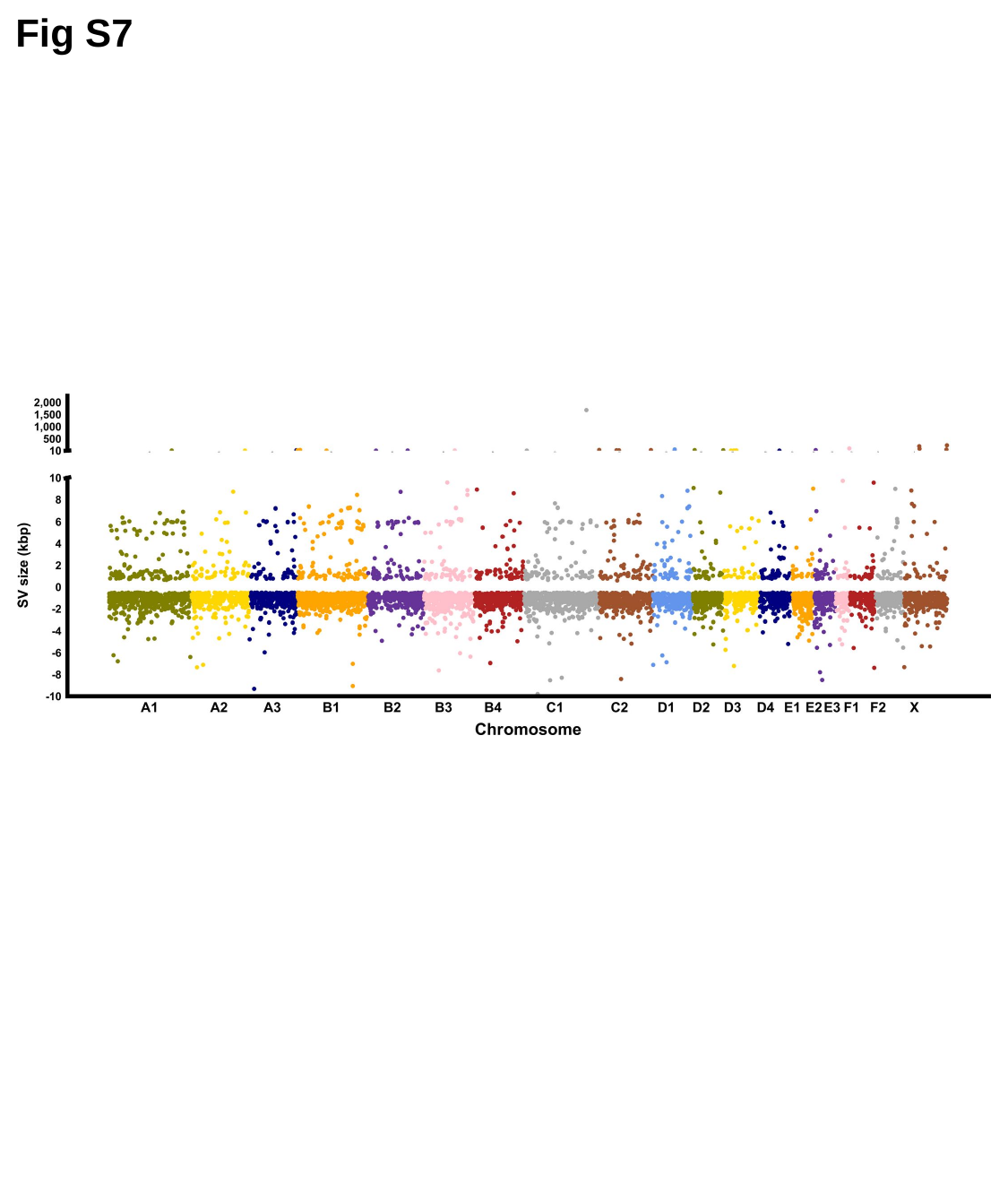

Fig S7

## Slide 8
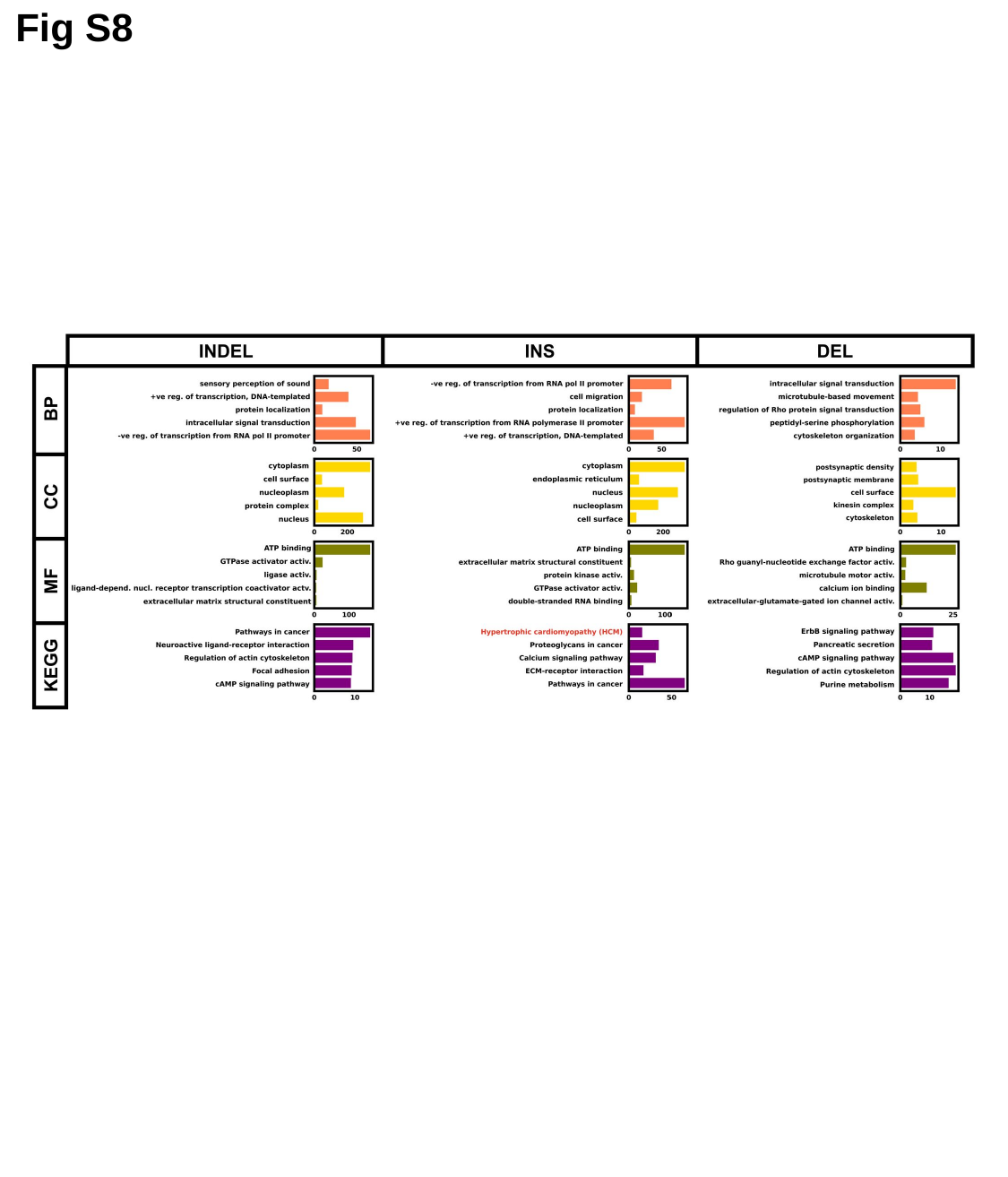

Fig S8

## Slide 9
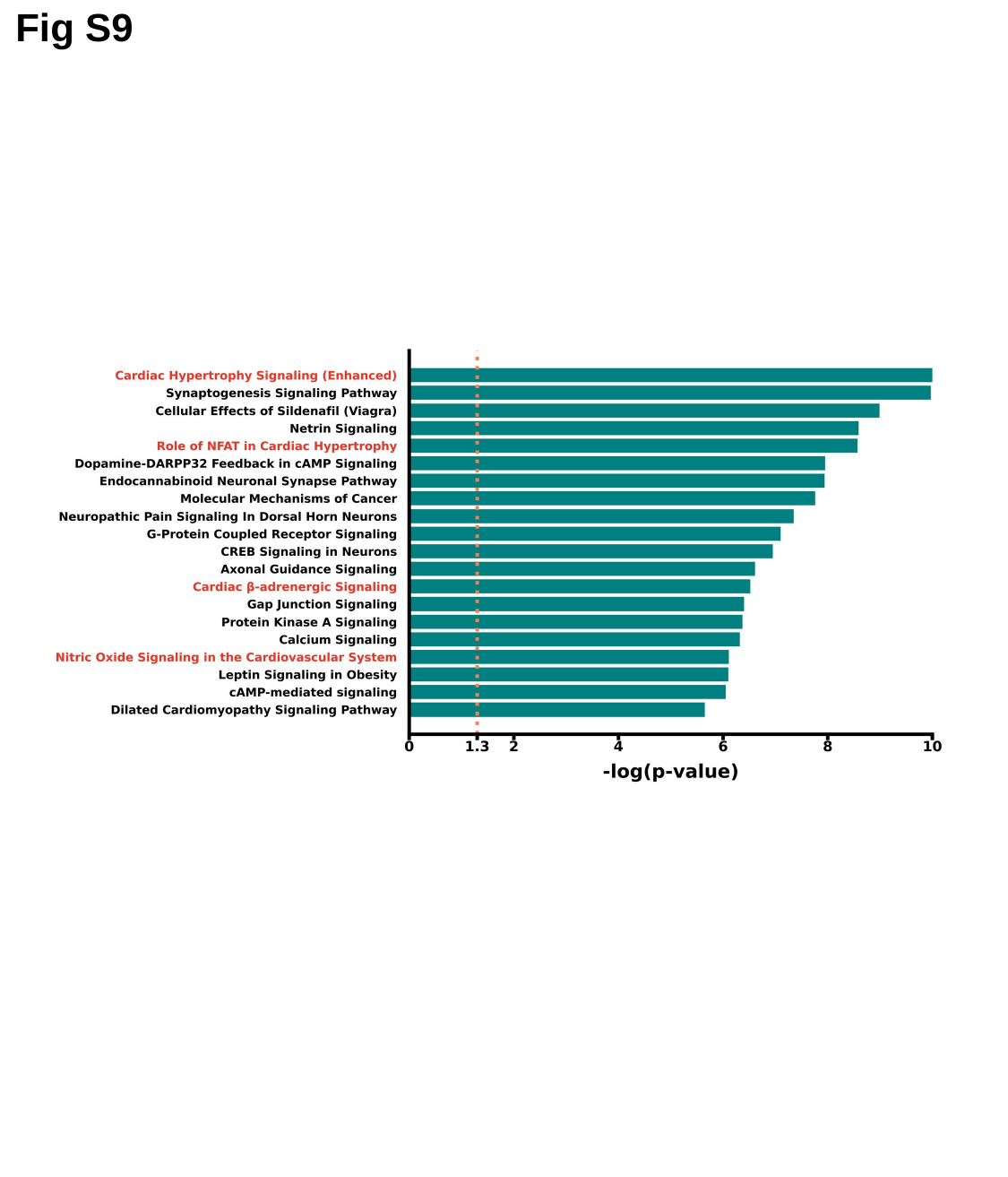

Fig S9

## Slide 10
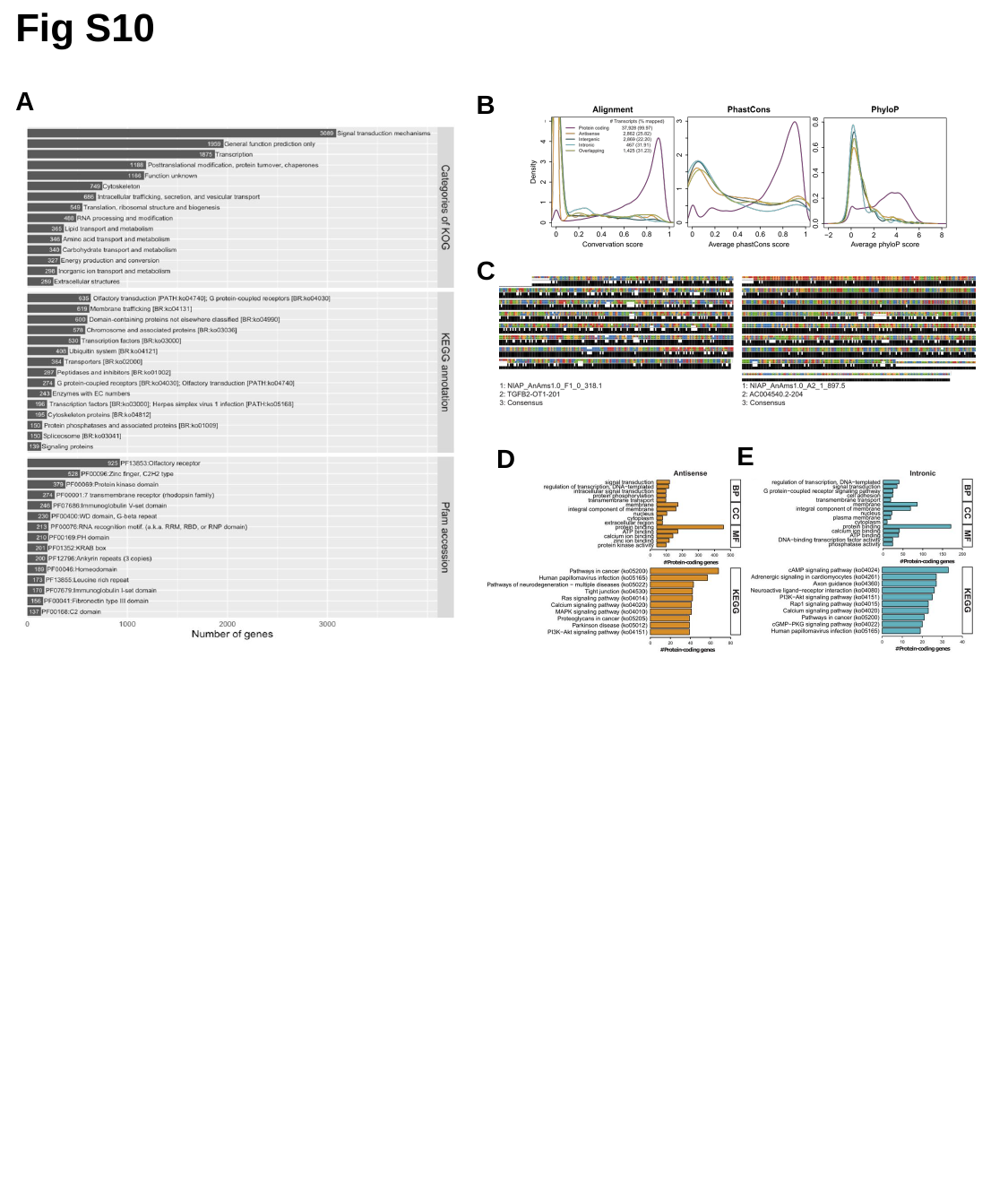

Fig S10
A
B
C
E
D

## Slide 11
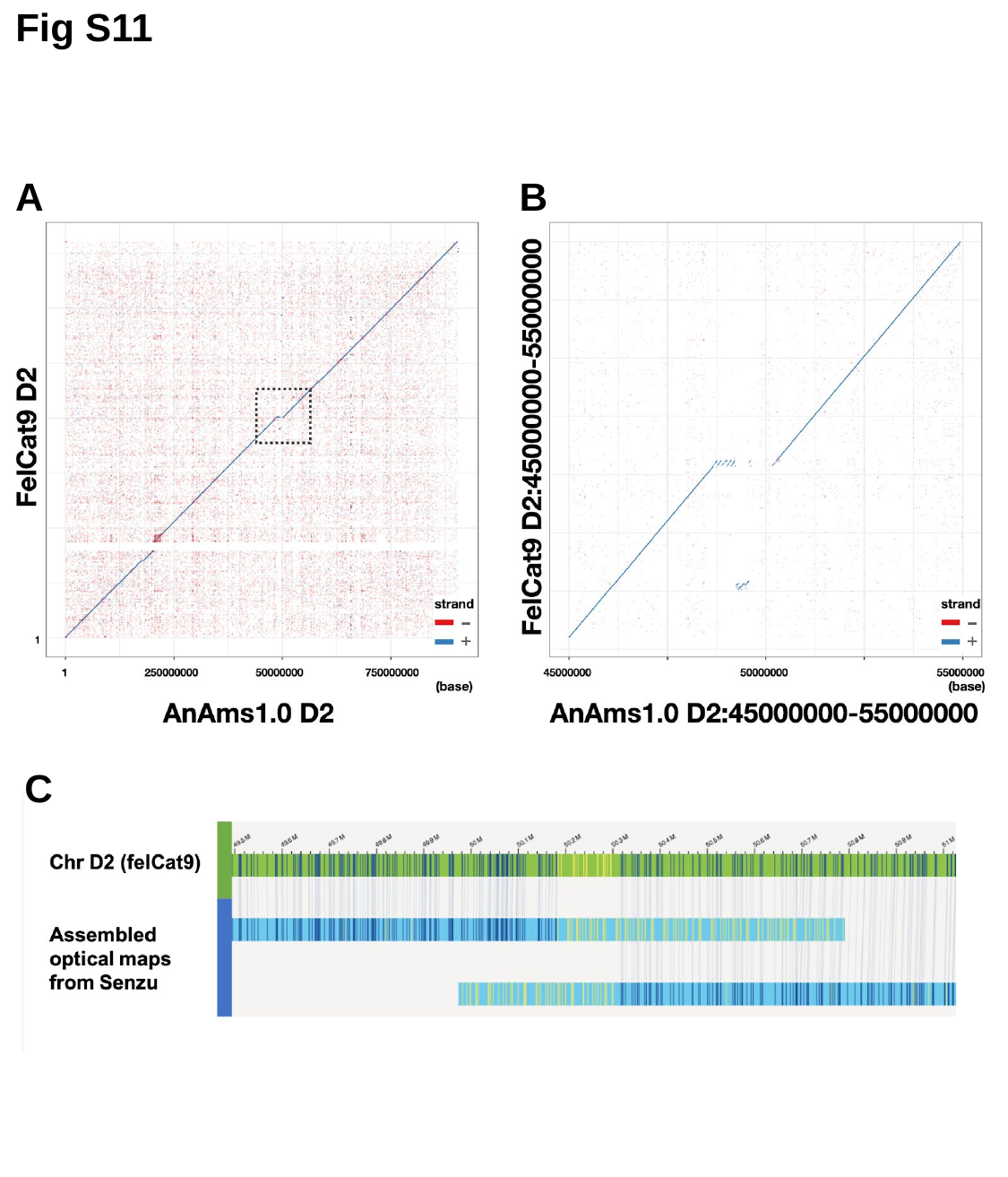

Fig S11
A
B
C

## Slide 12
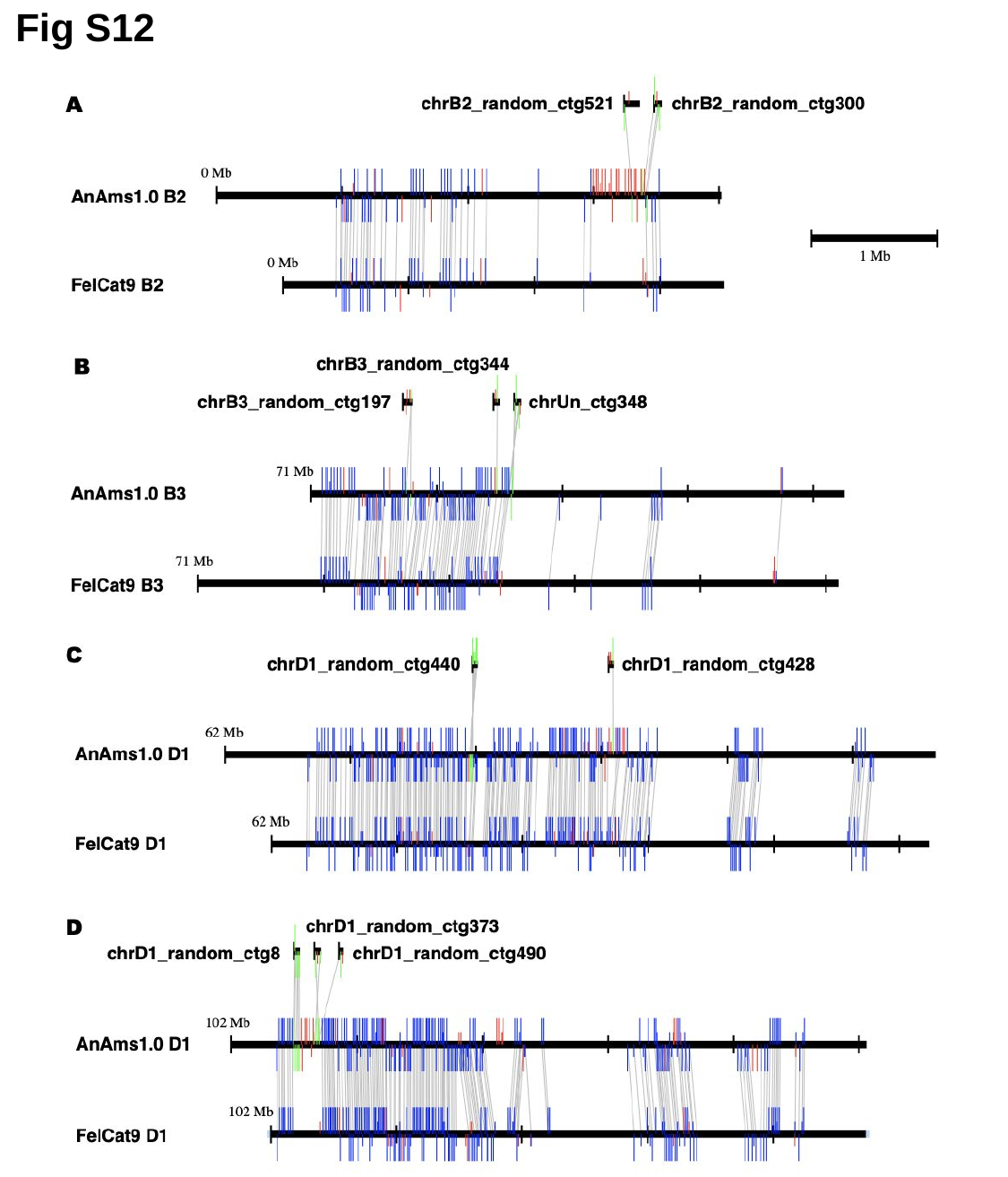

Fig S12

## Slide 13
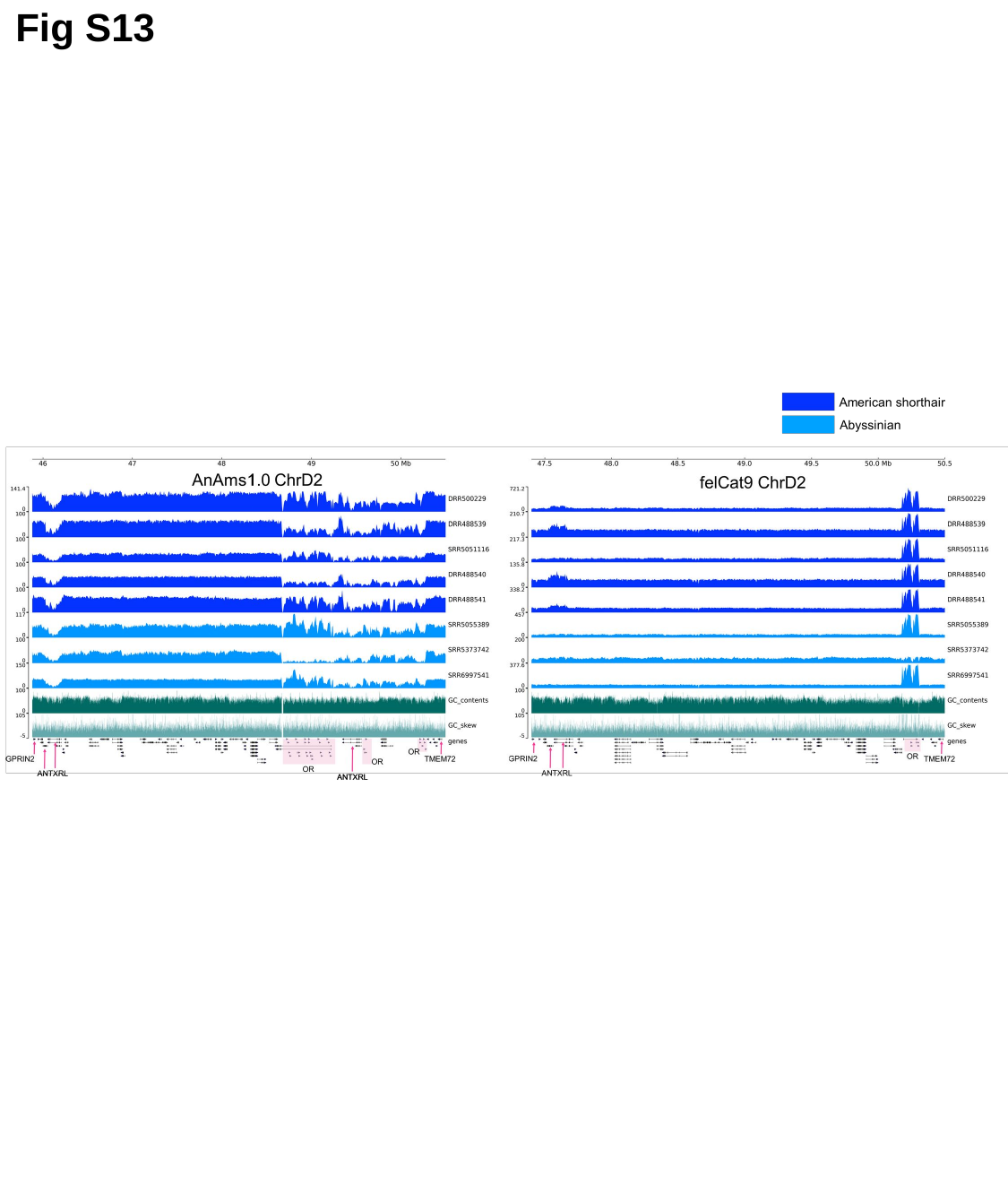

Fig S13
